# Characterization and use of the spent beam for serial operation of LCLS

**DOI:** 10.1107/S1600577515004002

**Published:** 2015-04-11

**Authors:** Sébastien Boutet, Lutz Foucar, Thomas R. M. Barends, Sabine Botha, R. Bruce Doak, Jason E. Koglin, Marc Messerschmidt, Karol Nass, Ilme Schlichting, M. Marvin Seibert, Robert L. Shoeman, Garth J. Williams

**Affiliations:** a Linac Coherent Light Source, SLAC National Accelerator Laboratory, 2575 Sand Hill Road, Menlo Park, CA 94025, USA; b Max-Planck-Institut für medizinische Forschung, Jahnstrasse 29, 69120 Heidelberg, Germany

**Keywords:** FEL, multiplexing, X-ray, serial femtosecond crystallography, SFX, nanocrystallography

## Abstract

X-ray free-electron laser sources such as the Linac Coherent Light Source offer very exciting possibilities for unique research. However, beam time at such facilities is very limited and in high demand. This has led to significant efforts towards beam multiplexing of various forms. One such effort involves re-using the so-called spent beam that passes through the hole in an area detector after a weak interaction with a primary sample. This beam can be refocused into a secondary interaction region and used for a second, independent experiment operating in series. The beam profile of this refocused beam was characterized for a particular experimental geometry at the Coherent X-ray Imaging instrument at LCLS. A demonstration of this multiplexing capability was performed with two simultaneous serial femtosecond crystallography experiments, both yielding interpretable data of sufficient quality to produce electron density maps.

## Abstract 2

Serial operation of FEL experiments was performed using the spent beam typically discarded after passing through a hole in a detector. The beam profile was characterized and results for two simultaneous experiments are reported.

## 1. Introduction   

X-ray free-electron lasers (FELs) are considered part of a broader family of light sources that make up the so-called fourth-generation light sources. FELs, however, by their very nature are very different from any previous synchrotron source and even from the other members of the fourth-generation class. They have a fundamentally different look with long linear facilities as opposed to the circular structures of synchrotron sources. This linear construction with single-pass acceleration of ultrashort electron bunches is at the source of the uniqueness and power of FELs, but the cause of the greatest frustration with them, which is the limited amount of beam time. In contrast to ring-based facilities which support multiple sources around the ring, linear-accelerator-based facilities can produce typically only one source at a time. Although new facilities will soon be capable of using multi-bunch modes and high repetition rates to distribute the electron pulses to multiple undulators and beamlines with fast kicker magnets, the number of sources and, therefore, the number of simultaneous experiments that can be performed at FEL sources will likely remain small for the foreseeable future.

X-ray FELs allow the generation of X-ray pulses with enough photons to produce measurable and interpretable signals from a single shot but that are also of ultrashort duration approaching or even reaching time-scales of electronic processes. For example, Auger decay life-times, typically on the order of a few to roughly 10 fs, are on the same time-scales as FEL pulse durations, which range between ∼5 fs and ∼100 fs. Furthermore, such short pulses can traverse the sample faster than the time-scales required for significant atomic motions, allowing snapshot data collection where the sample does not have time to move or change.

This realisation has led to the idea that radiation damage, which regularly presents a limitation in studying organic samples at continuous X-ray sources, could be greatly mitigated using the pulsed sources afforded by X-ray FELs. A single short pulse contains many more photons than would be necessary to cause irreparable damage to the sample using a time-integrating source focused to a micron-scale spot. All this incident energy, when the beam is tightly focused, leads to rapid sample charging and heating and ultimately the destruction of the sample. This process is, however, slower than the pulse duration leading to what is now known as the diffraction-before-destruction technique (Neutze *et al.*, 2000 ▶).

This approach gained immediate popularity at the first short-wavelength FEL user facility, FLASH in Hamburg, Germany (Ayvazyan *et al.*, 2002 ▶; Ackermann *et al.*, 2007 ▶; Tiedtke *et al.*, 2009 ▶). Properly utilizing FEL pulses can be challenging due to the damaging nature of the beam, which vaporizes any solid object in its path when tightly focused (Hau-Riege *et al.*, 2010 ▶). One must first pay close attention to optical and experimental design to avoid damaging the equipment (Soufli *et al.*, 2011 ▶, 2013 ▶; Bajt *et al.*, 2008 ▶). In addition, the sample itself as well as how it is delivered to the beam must be carefully planned. The sample will survive only for one pulse and efficient methods to renew it between every FEL pulse are often required. Many such techniques have been developed at FLASH (Chapman *et al.*, 2006 ▶, 2007 ▶; Bogan *et al.*, 2008 ▶; Barty *et al.*, 2008 ▶; Bostedt *et al.*, 2009 ▶), helping make the diffraction-before-destruction technique a reality.

Continued developments at the first hard X-ray FEL, the Linac Coherent Light Source (LCLS) (Emma *et al.*, 2010 ▶), have made the use of FEL sources and the diffraction-before-destruction technique more widespread. One of the most broadly used techniques at LCLS is serial femtosecond crystallography (SFX) (Chapman *et al.*, 2011 ▶; Boutet *et al.*, 2012 ▶), where a liquid jet (DePonte *et al.*, 2008 ▶; Weierstall *et al.*, 2012 ▶; Weierstall, 2014 ▶) containing small crystals is continuously flowed through the interaction region to provide fresh new protein crystals for each LCLS pulse. The technique is now one of the primary uses of the Coherent X-ray Imaging (CXI) instrument (Boutet & Williams, 2010 ▶; Liang *et al.*, 2015 ▶), with biologically relevant information now being obtained (Redecke *et al.*, 2013 ▶; Liu *et al.*, 2013 ▶). The SFX technique can take various forms such as fixed-target SFX (FT-SFX) (Hunter *et al.*, 2014 ▶), SFX using a lipidic cubic phase jet (LCP-SFX) (Weierstall *et al.*, 2014 ▶) and time-resolved SFX (TR-SFX) (Aquila *et al.*, 2012 ▶; Kern *et al.*, 2014 ▶; Kupitz *et al.*, 2014 ▶; Tenboer *et al.*, 2014 ▶). SFX techniques as well as other techniques commonly in use at the CXI instrument and LCLS, such as small- or wide-angle X-ray scattering (SAXS/WAXS) (Arnlund *et al.*, 2014 ▶; Sellberg *et al.*, 2014 ▶) and single particle coherent diffractive imaging (Seibert *et al.*, 2011 ▶), employ a constant stream of new samples that only interact weakly with the X-ray beam. The majority of the photons in the pulses do not interact with the sample and continue through a hole in an area detector, typically the Cornell–SLAC pixel array detector (CSPAD) (Blaj *et al.*, 2015 ▶; Hart *et al.*, 2012 ▶), used for forward-scattering measurements at CXI. This means that, for most of the experiments performed at CXI, the beam that has passed through the sample, the spent beam, contains close to 10^12^ photons that are simply dumped into either a diagnostic such as a fluorescent screen or even most of the time into a beam dump made of boron carbide.

Considering the limited amount of available beam time at a facility such as the LCLS, the idea of high-quality pulses terminating into a beam dump can seem inefficient. As part of an effort to increase access to the LCLS facility, which has involved the development of other multiplexing techniques such as the use of thin-diamond crystal monochromators (Feng *et al.*, 2013 ▶; Zhu *et al.*, 2014 ▶) and rapid mirror insertion (Yin *et al.*, 2013 ▶), developments were undertaken at CXI to refocus the spent beam to a second interaction region for use in an independent experiment. This article reports on the characterization of the refocused beam for one possible configuration of the CXI instrument as well as a demonstration of the serial operation of two experiments using this refocused or spent beam.

## 2. Experimental geometry   

**Figure 1:**
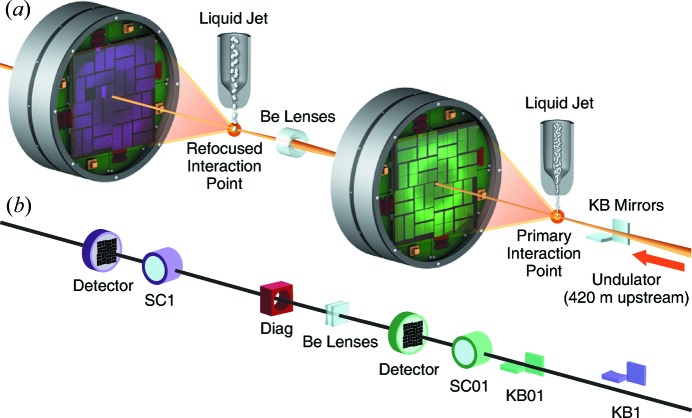
Experimental geometry for one possible use of the spent beam at CXI for serial operation of serial femtosecond crystallography (Serial-SFX, or S-SFX). The primary interaction point is at the location of the nanofocus produced by the CXI 100 nm KB system. The beam interacts with the primary sample and then diverges to the Be lenses used as refocusing optics downstream of the primary CSPAD detector. The beam is then refocused to the secondary interaction point, in the microfocus chamber of CXI. Relevant distances are given in Table 1 ▶. (*a*) Conceptual representation of the multiplexed experiments. (*b*) Schematic layout of the CXI beamline showing the microfocus system in purple and the nanofocus system in green. The microfocus mirrors (KB1) are not used for this geometry and are retracted out of the beam. A set of X-ray focusing lenses 1 m downstream of the nanofocus is used to refocus the beam to the microfocus sample chamber (SC1), with diagnostic elements such as a beam profile monitor (Diag) located between the lenses and the refocusing plane.

As described previously (Boutet & Williams, 2010 ▶) and concurrently (Liang *et al.*, 2015 ▶) in this issue of the *Journal of Synchrotron Radiation*, the CXI instrument was built with multiplexing in mind by having multiple interaction planes for the different focusing elements of the beamline. The two main focusing systems at CXI are a 1 µm Kirkpatrick–Baez (KB) system (Siewert *et al.*, 2012 ▶) which has focal lengths of 8.7 m and 8.3 m for the horizontally focusing mirror (HFM) and the vertically focusing mirror (VFM), respectively, and a 100 nm KB system with focal lengths of 0.9 m for the HFM and 0.5 m for the VFM. This is shown conceptually in Fig. 1 ▶(*b*) with the 1 µm (microfocus) system comprised of the 1 µm KB system (KB1), the 1 µm sample chamber (SC1) and the detector shown in purple; while the components of the 100 nm system, the 100 nm KB system (KB01), the 100 nm sample chamber (SC01) and its associated detector, are shown in green. The long focal length of the 1 µm KB system is required by the 420 m source distance and 30–60 µm FWHM source size in order to produce a FWHM focus in the order of 1 µm. This large focal length led to significant empty space along the beamline between the focusing optics and the 1 µm sample chamber. To optimize the limited floor space, this empty gap was filled by the entirety of the 100 nm (nanofocus) system shown in green in Fig. 1 ▶(*b*). The optical path of the microfocus beam from the mirrors to its focus passes completely by the nanofocus mirrors and focal plane [see Liang *et al.* (2015 ▶)]. In Fig. 1 ▶(*b*), the microfocus mirrors are shown out of the beam path as they are retracted when the nanofocus mirrors are in use. Conversely, when only the microfocus mirror system is in use, all components of the nanofocus system are removed from the beam path.

For typical operation of CXI, only one of these KB systems is in use at any given time. The different locations of the two mirror pairs as well as slightly different incidence angles mean that the beam axis is different for both systems and the entire beamline needs to be moved to change from using one system to the other. However, the serial arrangement of the focusing mirrors and the interaction planes allows naturally for multiplexing *via* the use of the spent beam from the nanofocus chamber, the most upstream of the two CXI sample chambers.

The experimental geometry is shown in Fig. 1 ▶(*a*). The two sample chambers can be used simultaneously by aligning the entire beamline on the 100 nm KB axis and utilizing the microfocus chamber for the secondary interaction point. The beam passing through the hole in the primary detector diverges until it intercepts a set of beryllium lenses that have been selected to properly refocus the beam of a desired photon energy. These lenses are mounted on an existing port on top of the detector chamber to locate them as close to the primary focus as possible, in this case 1 m. The system allows for three sets of lenses mounted to a single bracket that can be positioned transversely to the beam for proper centering of the lens clear aperture on the beam. Three different photon energies can then be used without any physical modification to the system, at the press of a few buttons.

**Figure 2:**
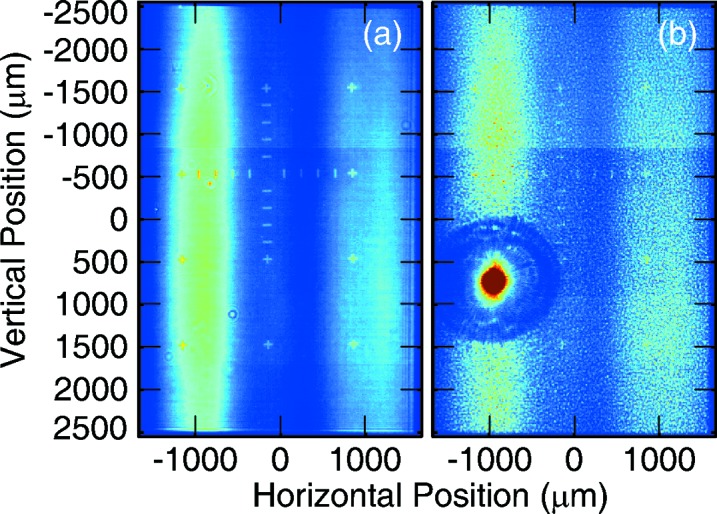
Beam profiles as measured on the Ce:YAG screen or profile monitor located roughly halfway between the Be lenses and the refocus plane. In both images shown, small lines and crosshairs are fiducials deposited on the Ce:YAG screen for resolution calibration. (*a*) Beam profile for the out of focus nanofocus beam after it is allowed to diverge for ∼3.1 m. These data were collected at 9.6 keV and show a magnified image of the pupil function of the nanofocus KB system at CXI. The two-lobe beam profile in the horizontal direction is caused by the limited size and the figure errors of the front-end enclosure offset mirrors (Barty *et al.*, 2009 ▶). The beam profile in the vertical is not distorted owing to the horizontal deflection of these front-end mirrors. (*b*) Similar image of the beam profile on the Ce:YAG screen captured with the Be refocusing lenses inserted. The limited aperture of the lenses as required to produce the short focal lengths needed causes some obvious aperturing of the beam for refocusing purposes. Only the part of the beam within the circular area in the bottom left is refocused. The rest continues to diverge and is removed by using slits downstream of the lenses. The lens aperture is also seen to be wider than a single lobe of the horizontal beam profile which has an impact on the size of the refocused spot by effectively reducing the numerical aperture of the system. For both images shown, the beam profile extends almost to the edges of the plotted area where a sharp cutoff can be seen due to the finite nanofocus KB aperture.

The relevant components and their relative distances are listed in Table 1 ▶. Shown in Fig. 1 ▶(*b*) is a beam profile monitor (labeled Diag) which consists of a fluorescent screen that can be inserted between the two interaction points to characterize the beam profile downstream of the refocusing lenses but prior to reaching the secondary focus. Images from this screen are shown in Fig. 2 ▶.

## 3. Refocused beam simulations   

Geometrical optics can be easily used to calculate the expected beam profile at both the primary focus and the refocused plane. Using the calculated source size and location of LCLS, 30–60 µm FWHM (depending on the photon energy) and 420 m, respectively, one can calculate the focus sizes and location assuming perfect optics by using the thin lens equation 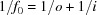, where 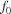 is the focal length of the optical element, *o* is the object distance from the optical element and *i* is the distance of the image from the optical element. The image magnification is given by 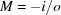. This will give a reasonable estimate of the primary spot size produced by the 100 nm KB mirrors at CXI owing to the achromatic nature of KB mirrors and the large size of the KB optics which, while not capturing the full beam, come sufficiently close to doing so for this simple calculation to be reasonable. Application of the thin lens equation produces a ∼70 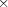 120 nm FWHM primary focus, with the beam being smaller in the vertical due to the shorter focal length of the vertically focusing mirror. This spot size is an estimate based on ideal optics and an ideal source, both of which are approximations. In particular, the actual source size and location of the LCLS source are unknown. Nevertheless, for the KB beam, these values are generally consistent with the observed focus size of approximately 200 nm FWHM in the nanofocus chamber.

However, continued use of geometrical optics calculation for the refocused beam will give a significantly wrong answer for the presented geometry for two primary reasons. First, the Be lenses used for refocusing are chromatic with the focal length 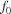 varying with 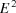, where *E* is the photon energy. The LCLS beam has a typical bandwidth of 0.2% FWHM which usually does not represent an issue when using lenses as the primary focusing element. Under such usual circumstances, Be lenses are used to focus the incident LCLS beam with a source distance much larger than the distance to the focus. This is a demagnifying optical system which creates at the focus an image of the source that is smaller than the source. As can be obtained from the thin lens equation, under the condition that 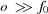, the location of the image is not so sensitive to the source position or even to small changes in 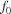. In the ultimate limit of the source at infinity, the image is located exactly at 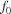 and small changes to the source point have no impact on the image location. For chromatic optics, this also means that small changes in 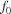 with photon energy cause the focal plane for different energies to move linearly with 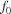. Under typical use, for micrometer-sized foci at LCLS, this means the focal plane changes within the energy bandwidth at LCLS are of the same order as the depth of focus of the beam, leading to no significant increase in the spot size. This is no longer true if one tries to use Be lenses or other chromatic optics for producing a nanofocus, where a monochromatic beam is required (Schropp *et al.*, 2013 ▶) in order to avoid significant smearing of the beam profile owing to different photon energies focusing at different planes.

The refocusing system used here is, however, a magnifying system, where the image distance is larger than the source distance (4.7 m *versus* 1 m). In the same way a demagnifying system keeps the image at a similar position for large changes in the source location, a magnifying system will move the image significantly for small changes in the source location, or equivalently for chromatic optics, small variations in the focal length of the optics. Therefore, chromaticity plays an important role in the configuration presented here. A simple application of geometrical optics ignoring chromaticity for the experimental geometry presented here would produce a ∼350 nm 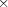 600 nm FWHM at the refocus position, which is incorrect by a significant amount.

The second reason why the geometrical optics calculation fails in the presented case is due to the large divergence of the nanofocus beam which greatly overfills the Be lens aperture, as can be seen in Fig. 2 ▶. The CXI nanofocus beam has a divergence of 1 mrad 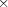 2 mrad leading to a magnified image of the KB pupil function at the Be lenses, where the 1.1 mm 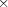 1 mm aperture of the mirror is magnified to 1.2 mm 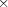 2 mm. The lenses required to refocus hard X-rays with the short focal length required here have clear apertures of ∼450 µm in diameter, leading to the significant aperturing of the beam as shown in Fig. 2 ▶. This reduces the numerical aperture of the optical system, making the focus larger due to significant diffraction at the refocused plane. It also causes a reduction in the number of photons refocused due to aperturing. A finite aperture with a coherent beam causes a double loss in power density at the focus by reducing the number of photons and increasing the spot size.

**Figure 3:**
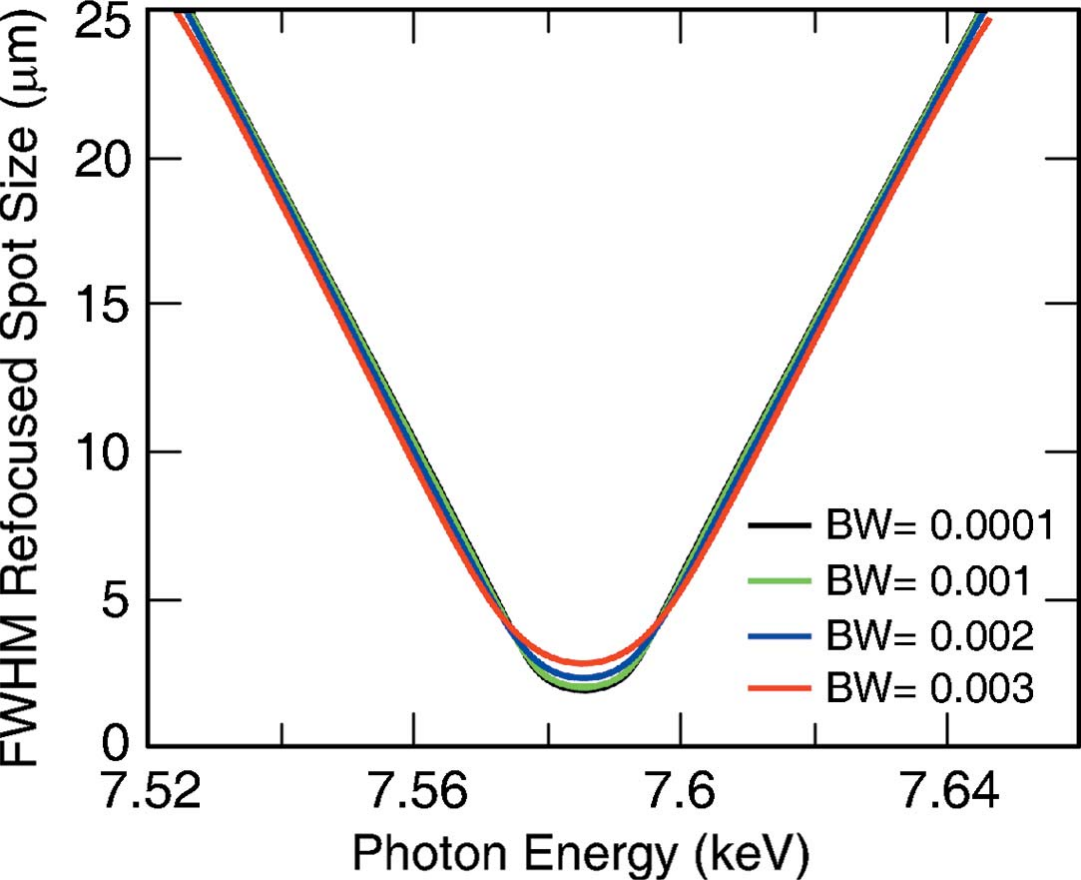
FWHM spot size of the refocused beam at the secondary interaction plane calculated for a Gaussian spectral bandwidth (BW) at LCLS between 0.0001 (0.01%) and 0.003 (0.3%). This was obtained by performing wave propagation calculations from a Gaussian source through the multiple focusing optics to the refocused plane. This was performed at multiple energies and then incoherently summed over the spectral bandwidth. The process was repeated for different central photon energies while keeping the bandwidth constant for each of the curves.

In order to properly simulate the expected refocused beam profile, wave optical simulations were performed from the LCLS source point to the refocused plane, passing through the KB mirrors and the Be lenses, and taking into account real optical aperture sizes. This was performed at multiple photon energies within the expected bandwidth of LCLS around a central energy that is properly refocused at the second interaction plane. Then a Gaussian spectral profile was used to perform an incoherent sum of all simulated beam profiles with proper weighing for a given bandwidth. This was repeated for multiple central photon energies and for multiple bandwidths. Simulations were performed at 6.8 keV, 7.6 keV and 9.6 keV representative of the lens configurations of four, five and eight lenses of 50 µm radius, respectively, that were used for the experiment. Shown in Fig. 3 ▶ are the results for a bandwidth of 0.2% at ∼7.6 keV.

Keeping the bandwidth constant but scanning the central energy produces a clear focus of ∼2.5 µm FWHM. The beam, as expected, becomes bigger as the central photon energy moves away from the optimal value. As the LCLS source size does not vary too significantly with photon energy over the range used at CXI (from 5 keV to 10 keV), to first order, the behavior of the refocused beam size is similar for all photon energies used as long as a Be lens stack with suitable focal length is used. Therefore, for the experimental geometry presented here and an LCLS self-amplified spontaneous emission (SASE) beam, a minimum spot size of 2.5 µm FWHM should be expected, with significant sensitivity to energy fluctuations which can occur from day to day, likely requiring checking the focus periodically. Also playing a role in the optimal photon energy for refocusing is the tuning of the machine and the beam divergence from the source on any given day, which may be sufficiently different to slightly change the focal plane. The use of a self-seeded beam (Amann *et al.*, 2012 ▶) with its more monochromatic spectrum could be used to produce a smaller refocused beam, at the cost of lost intensity. Examples of the simulated beam propagation simulations can be found in the supporting information.

To summarize the simulations, the calculated beam parameters are for the primary focus: 70 nm 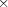 120 nm FWHM with a 60–70% transmission from the source. For the secondary focus: 2.5 µm 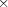 2.5 µm FWHM with a 4–6% transmission from the initial LCLS source (6–8% efficiency in the refocusing stage). The range of parameters is a consequence of the varying divergence and source size for different photon energies. This is under the assumption of a Gaussian beam with perfect optics of limited sizes. In practice, the beam in not quite Gaussian and optics are not perfect, leading to possibly reduced efficiency and increased spot size for the nanofocus and subsequently to the refocused beam.

## 4. Refocused beam characterization   

The refocused beam profile was measured on a Ce:YAG screen at the sample location in the microfocus chamber and imaged using a long-range microscope. The 2.5 µm pixel size of the image on this microscope was characterized using an arrayed pattern. The long working distance of this microscope leads to a relatively small numerical aperture and blurring of the image of the beam profile. Gaussian two-dimensional profiles were fitted to each of the images as the electron beam energy was scanned around a central value. The results were sorted, based on the electron energy for each shot, and then converted to a value for the photon energy for any given pulse. The width of the fitted beam profiles for both the horizontal and the vertical direction were obtained for each pulse by deconvolving the fitted Gaussian with the point spread function of the microscope, which was determined from the known geometry of the optics and the working distance used.

**Figure 4:**
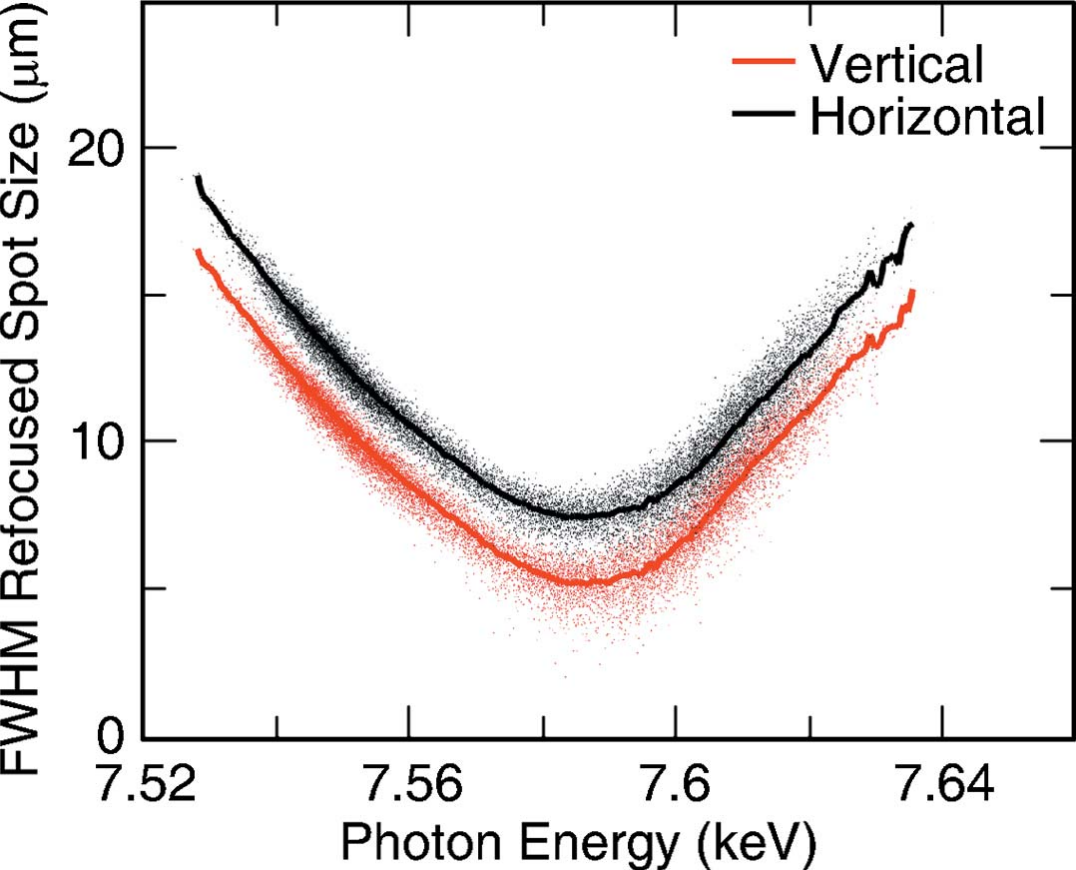
Refocused beam profile characterization at ∼7.6 keV. The results are shown on the same scale as the simulations of Fig. 3 ▶ for ease of comparison. The results are shown in red for the vertical profile and in black for the horizontal profile. Each of the small colored dots represents an LCLS shot to which a two-dimensional Gaussian profile was fitted to the measured profile. The solid lines represent the average of the fitted spots within certain central photon energy bin. The slope at which the FWHM increases with deviations from the ideal photon energy is smaller than expected from simulations. This could be an indication that the spectral bandwidth of the beam was larger than expected. The beam profile at the optimally focused energy is fairly consistent with the expected 0.2% bandwidth beam with a Gaussian source propagating through the CXI beamline. A larger measured spot size than simulated is not so surprising given that perfect optics were used for the simulation. The data are consistent with both expectations of larger foci due to optical aberrations and a potentially larger than expected bandwidth but do not allow us to claim one over the other.

**Figure 5:**
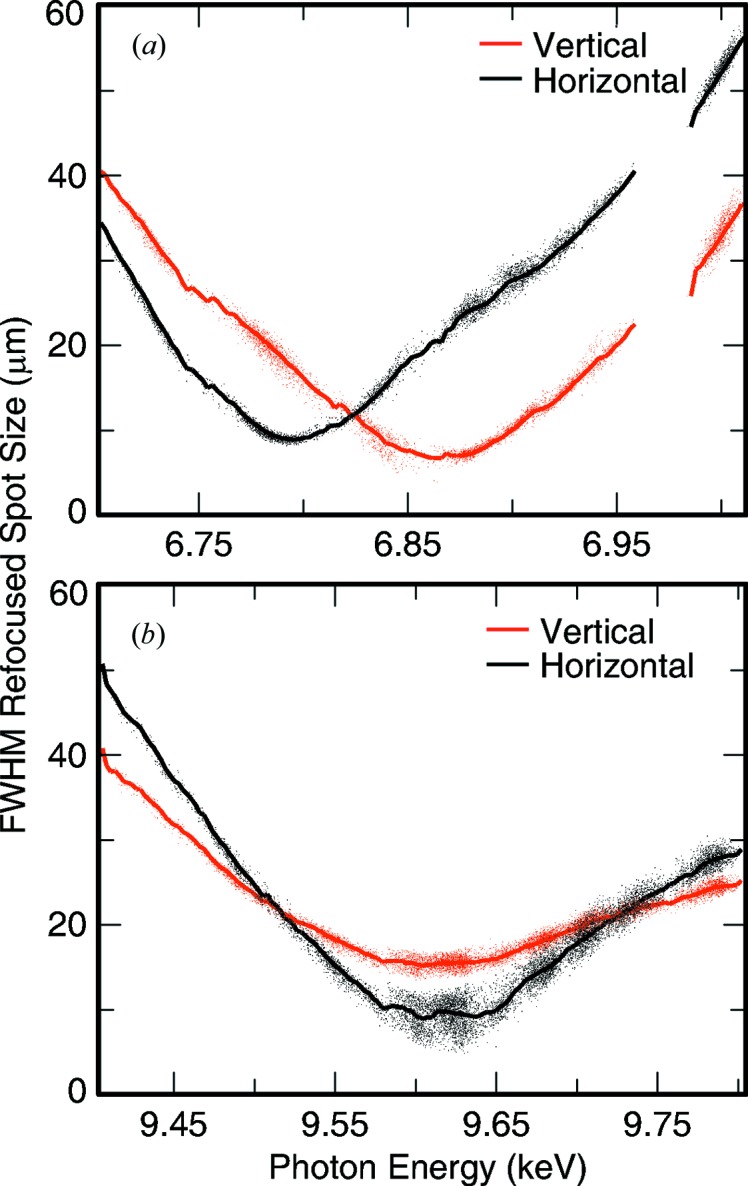
FWHM of the refocused beam profile for (*a*) 6.8 keV and (*b*) 9.6 keV. (*a*) The results show a clearly astigmatic beam due to misaligned lenses in the horizontal leading to a weaker total parabolic shape and a weaker lens in the horizontal direction. (*b*) The reason for the significantly larger than expected size in the vertical direction is unclear.

This was performed at three different photon energies, for each of the three lens stacks installed at the time of the experiment. The results for ∼7.6 keV are shown in Fig. 4 ▶. Other energies showed similar results with a few exceptions, as shown in Figs. 5 ▶ and 6 ▶. For example, the lens stack for 6.8 keV was clearly misaligned in the horizontal direction with the four lenses used not on the same optical axis. This was clearly visible on the Ce:YAG screen between the two foci, similar to Fig. 2 ▶, where the shadow of the lens showed multiple non-concentric circles, leading to a weaker lens in the horizontal direction. This produced an astigmatic focus for this lens stack with the vertical direction focusing at higher energy by 75 eV, as shown in Fig. 5 ▶.

Also investigated were the day-to-day fluctuations of the focus. The same energy of 7.6 keV was used on multiple days and the electron energy scan was repeated each day. The best focus was found to vary by up to 30 eV from day to day with the focus size also varying from 6 to 8 µm. The astigmatism also varied from day to day. This is most probably due to slightly different divergence of the beam from machine tuning as well as differences in beam alignment at CXI. As can be seen from Fig. 2 ▶, the beam profile at the refocusing lenses is not round due to aperturing and figure errors of the upstream hard X-ray offset mirrors system (HOMS) (Barty *et al.*, 2009 ▶). The beam profile at CXI depends heavily on the exact alignment of these mirrors, leading to different profiles on any given day and, therefore, different illumination of the limited aperture of the refocusing lenses. On some days, the two-lobed beam profile seen in Fig. 2 ▶ can instead have three clear lobes. The refocused beam profile at any given photon energy would be affected by the exact incident beam profile that enters the CXI hutch and which is not fully reproducible. Also, it is quite likely that the lenses may not be placed exactly at the same location every time relative to the beam profile, leading again to different illumination and different focusing properties. The CXI hutch is very far away from the source and there is only limited beam pointing capabilities, with a single motor step on the HOMS mirror angle displacing the beam at CXI by roughly a quarter of its size. It is, therefore, very difficult to reproduce the exact same alignment every day. All beam characterization results including daily fluctuations are shown on Figs. 5 ▶ and 6 ▶.

Finally, the efficiency of the refocusing was measured by integrating all the intensity of the full beam profile shown on Fig. 2 ▶ and comparing that with the integrated intensity within the clear shadow of the lens. If was found that the limited aperture of the lenses leads to a 12% transmission to the refocusing plane. This was measured at both 9.6 and 7.6 keV, but no such measurement was made at 6.8 keV. The wave optical simulations, which also included Be absorption losses, suggested an 8% efficiency depending on the photon energy. This is the efficiency of the refocusing only, which does not include other upstream losses from the front-end mirrors as well as the KB mirrors. Together, these represent another factor of ∼50% transmission losses. Thus, for the experimental geometry presented here, with a pulse energy of 3 mJ at the source, one can expect ∼1.5 mJ at the primary interaction point and ∼240 µJ at the refocused interaction point.

The discrepancy between the measured and calculated efficiency can, in part, be explained by the presence of a stronger lobe in the real beam profile at the plane of the Be lenses. The lenses were aligned to the stronger lobe, making the efficiency higher by cutting away the weaker part of the beam. The simulations were performed with a beam symmetric around the origin in both directions as can be seen in the supporting information.

An upper bound for the shot-to-shot positional jitter of the refocused beam can be obtained from the central position of the fitted Gaussian profiles for every pulse. The distribution of the beam center was obtained and showed a Gaussian profile with a 2.3 µm r.m.s. fluctuation, a scale similar to the refocused beam size itself. It must be pointed out, however, that the central position of the measured beam is subject to not only real beam motions and beam pointing fluctuations but also vibrations in the optical system used to view the beam profile. Evidence indicates that the camera system used here is the main source of the vibrations observed, with the beam itself moving less than the observed motion of the image. However, beam position fluctuations representing an appreciable fraction of the beam size (∼20%) are expected with LCLS and FEL and the upper bound obtained here, including vibrations of the imaging system, are consistent with the expectations.

## 5. Serial operation of two experiments   

The characterized refocused beam using the ∼7.6 keV lens stack was used to perform two simultaneous and independent experiments within CXI using the geometry shown in Fig. 1 ▶. Two serial femtosecond crystallography experiments, both using gas dynamic virtual nozzles to deliver a stream of hydrated crystals to the respective interaction regions, were set up. Both were operated independently from the same control room using independent data acquisition systems.

Data were collected from lysozyme microcrystals, using smaller crystals (∼1 µm 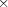 1 µm 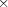 2 µm) in the nanofocus chamber to avoid detector saturation despite the use of the full beam intensity. Larger crystals (∼3 µm 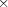 3 µm 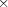 5 µm) were used in the refocus chamber because of the expected larger beam size as well as the limited efficiency of the re­focusing leading to significantly reduced power on the sample. Crystals were prepared as described by Boutet *et al.* (2012 ▶), with changes in protein concentration to control crystal size. A 2.5 ml lysozyme solution with pH 3.0 (36 mg ml^−1^ for the larger crystals, 56 mg ml^−1^ for the smaller crystals) was mixed rapidly with 7.5 ml precipitant solution (20% NaCl, 6% PEG 6000, 1 *M* Na acetate pH 3.0) and left overnight at 277 K on a vertically rotating turntable. After settling of the crystals, the crystal growth solution was exchanged several times for storage solution (8% NaCl, 0.1 *M* Na acetate pH 4.0). Before data collection, the crystalline slurry was filtered using a 20 µm stainless steel inline filter. The final sample contained approximately one-third solid settled crystalline material by volume.

For the primary data set collected with the nanofocused beam, *CASS* (Foucar *et al.*, 2012 ▶) was used to identify hits as described previously by Barends *et al.* (2014 ▶, 2013 ▶). The CSPAD used for this data set was behaving normally, with noise characteristics as expected for a typical CSPAD and allowed for this standard method to be used.

For the refocused data set, a different approach was required because of the poorer characteristics of the second detector used for this sample chamber. This detector had an out of the ordinary large number of bad (hot) pixels easily confused with signal. Also, the Bragg reflections in the re­focused data set encompassed fewer pixels and were weaker in comparison with the primary data set. Therefore, a later version of *CASS* was used to identify hits with the procedure modified to include a new feature called PostProcessor with ID 208 (Foucar, 2015, in preparation) to identify possible Bragg reflections on the offset-corrected and bad-pixel-masked images. A set box size of 4 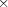 4 pixels, which results in a box size of 9 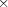 9 pixels and a threshold of 400 ADU, was used to identify images containing hits.

Because of the different characteristics of the two detectors, the settings for bad pixel identification needed to be different. While for the upstream detector a fixed upper threshold of 4.3 ADU was sufficient, the noise characteristics of the downstream detector were unusually bad. Therefore, an additional lower boundary needed to be included. The range for which noise was acceptable was set from 2 to 7 ADU. Pixels with noise values greater or less than this range were masked as bad.

The additional threshold for bad pixel identification and the different approach on identifying hits were essential for successful analysis in the downstream experiment as otherwise the fewer hits found with the same approach could not be indexed. This was not due to the refocused beam itself but rather to the detector characteristics, likely caused by accumulated damage to pixels over months of operation (Tomada *et al.*, 2012 ▶).

*CrystFEL* (White *et al.*, 2012 ▶) was used for Monte Carlo integration of the data (Kirian *et al.*, 2010 ▶, 2011 ▶). Data collection statistics are reported in Table 2 ▶. The lysozyme structure determined using 40 fs FEL pulses [PDB entry 4et8; Boutet *et al.* (2012 ▶)] was refined against both data sets followed by two cycles of iterative rebuilding and refinement, using *COOT* (Emsley & Cowtan, 2004 ▶) and *REFMAC5* (Murshudov *et al.*, 2011 ▶) and riding hydrogen atoms, resulting in models of excellent quality. Refinement and model statistics are reported in Table 3 ▶. The structures and structure factor amplitudes have been deposited in the Protein Data Bank as entries 4rw1 and 4rw2.

**Figure 7:**
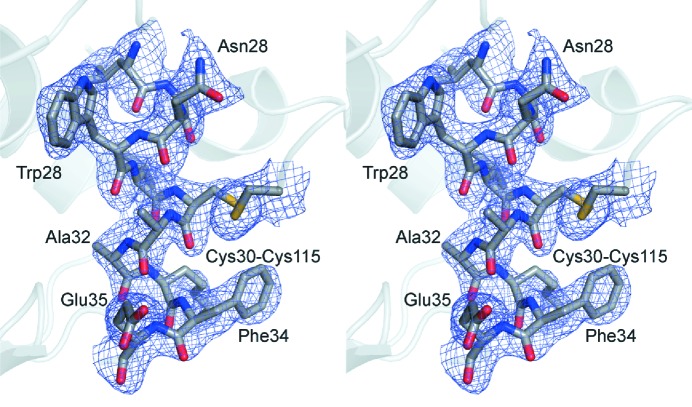
Stereo image, showing part of the simulated annealing composite omit map calculated using *PHENIX* (Adams *et al.*, 2010 ▶) for the data from the refocused beam, contoured at 1.0σ and overlaid on the final refined structure.

Importantly, the electron density maps for the structure determined using the refocused beam clearly showed the positions of extra water molecules and even a dual conformation for the side chain of Gln121. Moreover, the simulated annealing composite omit map for this structure is of excellent quality too, as shown in Fig. 7 ▶. Taken together, these results demonstrate that the refocused spent beam produced with the presented geometry allows high-quality SFX data to be collected from relatively large, well diffracting crystals, even despite the very poor efficiency of the refocusing system used in this demonstration. The reduced resolution of the data collected in the microfocus chamber is most likely caused by both the crystal quality, as showed by the Wilson plot, and the low efficiency of the refocusing system leading to a reduced signal and signal-to-noise ratio.

## 6. Conclusions   

The beam profile of the spent beam using the CXI nanofocus as the primary focusing element was simulated and characterized. The results are reasonably consistent with the simulations, indicating that an ∼5 µm FWHM spot should be expected as the best-case scenario for this particular geometry. This geometry was used as a proof-of-principle using equipment that was available as part of the base CXI system. However, it is very clear that this is far from an optimal geometry to re-utilize the spent beam. The nanofocus KB system produces a beam that is much too divergent to make efficient re-use of the spent beam. The Be lenses cannot be placed close enough to the primary focus to allow efficient capture of the spent beam, leading to multiple issues such as limited efficiency. Furthermore, the need to place the Be lenses as close as possible to the nanofocus to maximize efficiency leads to a long distance from the lenses to the refocused plane, making the system even more chromatic due to the magnifying optical configuration. But at the time of the experiment, this geometry was the only available option at CXI. It was still sufficient to demonstrate the concept and to allow two simultaneous experiments to take place. It allowed the demonstration of serial SFX (S-SFX) yielding two simultaneous interpretable data sets from the same LCLS pulses.

It also validated simulations that will be used to estimate the refocused beam size for a new system under development at CXI, the serial sample chamber (SSC). It will allow the very same concept presented here to be used for the microfocus KB system instead of the nanofocus KB system (Liang *et al.*, 2015 ▶). A new sample chamber will be added between the two existing detectors that can be used with the microfocus system. The same Be lens system will be used as presented here, with the lenses 1 m downstream of the microfocus. The microfocused beam has ten times less divergence than the nanofocused beam and, therefore, the beam at the lenses will be smaller than the clear aperture, leading to a much more efficient refocusing system and removing issues of overfilling optics that make the beam larger at the refocused plane. The SSC system will also have a roughly one-to-one imaging system, making chromaticity less of an issue. Overall, it is expected that the refocused beam at the SSC system will be a much more efficient and usable option for many ‘parasitic’ experiments at CXI. The system is expected to be commissioned in the middle of 2015 and should be made available to users soon after. The SSC system at CXI is one of the many ways in which LCLS hopes to add beam time to relieve the oversubscription in the system.

A key challenge, however, exists in performing challenging experiments in series in the same hutch, where access to one experiment, for example, to change samples or exchange a clogged sample nozzle, takes the beam away from the other experiment as well. In order for the spent beam use to be practical and efficient, it will be required that the two experiments performed be relatively smooth running because unforeseen downtimes will not coincide which could, in practice, double the downtime for both experiments. To minimize this issue, the primary experiment is expected to be in full control of access to the hutch, with hutch access to the secondary experiment only coinciding with a need to do so for the primary experiment. In this regard, the secondary experiment will be entirely parasitic and required to be relatively simple with limited needs for access. For example, the nanofocus spent beam configuration presented here has also been used for a parasitic experiment involving the use of fixed targets, where sample delivery issues are not expected to cause downtime. This is an example of a simple experimental geometry that can make good use of the spent beam. The most challenging LCLS experiments are expected to be performed in the primary beam, with only less-demanding measurements having the option of parasitic beam use. In the case of simultaneous SFX measurements with liquid jets, the experiments will be at the mercy of the reliability of the jets, with parasitic operation suffering the consequences of issues with both jets. For example, during the measurements presented here, a new nozzle design used and its associated growing pains led to significant downtime in both experiments, making the running time of the second experiment small (20%). While it is expected that SFX experiments will continue to become more reliable, simple measurements only requiring a small amount of beam time such as protein crystal screening could be best suited to this mode of operation.

## Supplementary Material

Examples of the simulations performed. DOI: 10.1107/S1600577515004002/yi5010sup1.pdf

## Figures and Tables

**Figure 6:**
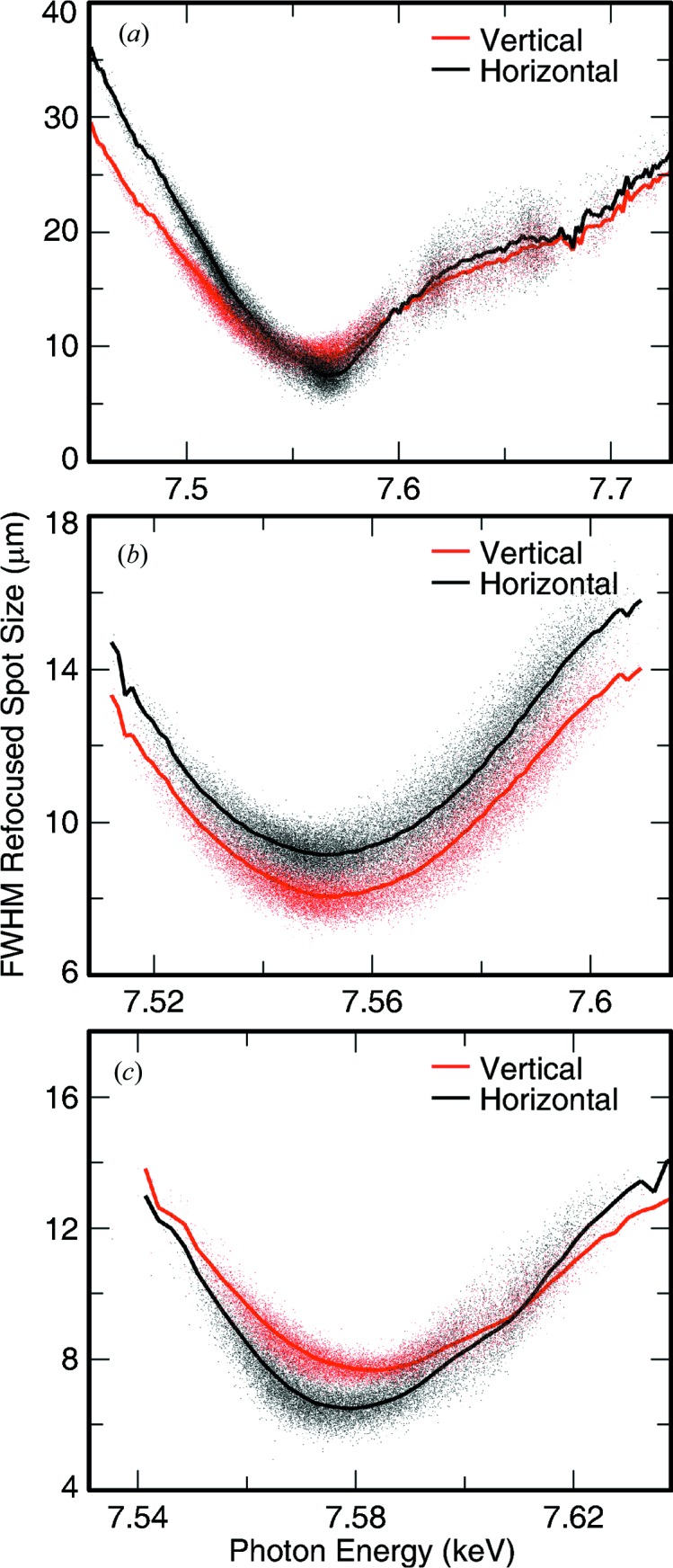
Day-to-day fluctuations of the refocused beam profile at 7.6 keV. (*a*), (*b*) and (*c*) were obtained at different times during the multi-day beam time performing a similar electron energy scan on different days. The minimum spot size as well as the optimally focused photon energy vary from day to day. The spot size dependence on photon energy also changes and the relative size of the horizontal and vertical profiles is not maintained. Differences in tuning and alignment on any given day are likely at the source of these fluctuations.

**Table 1:** Optical element locations

Optical element	Location (m)	Distance to previous element (m)
100nm HFM	0.900	
100nm VFM	0.500	0.400
Primary interaction point	0.000	0.500
Be lenses	1.000	1.000
Beam profile monitor	3.100	2.100
Refocused interaction point	4.746	1.646

**Table 2:** SFX data collection statistics

	Primary focus (nanofocus)	Refocused beam
Space group	*P*4 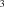 2 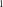 2	*P*4 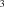 2 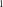 2
*a*, *b*, *c* ()	79.2, 79.2, 38.2	79.0, 79.0, 38.2
, , ( 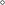 )	90, 90, 90	90, 90, 90
No. of images	1123382	543802
No. of hits	25647	74584
No. of indexed images	12894	9649
Indexing rates (%)	50.2	12.9
Resolution range ()†	401.9 (2.01.9)	402.3 (2.42.3)
Completeness (%)	100 (100)	100 (100)
Multiplicity	454 (114)	339 (94)
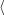 *I*/(*I*) 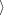	6.4 (1.57)	4.9 (1.2)
*R*_split_ ‡	0.112 (0.625)	0.167 (0.728)
CC	0.984 (0.409)	0.947 (0.562)
CC*	0.996 (0.762)	0.986 (0.848)
Overall *B* factor ( 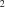 )§	41.5	67.7

† Values in parentheses are for the highest resolution shell.

‡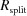 = 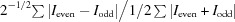 (White *et al.*, 2012 ▶), where 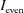 and 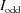 are intensities determined from all even and all odd numbered images, respectively.

§ It is likely that these Wilson *B*-factors are artificially high due to the inclusion of weak diffraction data at high resolution from crystals with poorer diffraction strength than average.

**Table 3:** Refinement statistics

	Primary focus (nanofocus)	Refocused beam
PDB entry	4rw1	4rw2
Cutoff	None	None
No. of reflections, working set	9531	5451
No. of reflections, test set	517	291
Final *R*_cryst_	0.219	0.204
Final *R*_free_	0.256	0.251
Cruickshank DPI	0.191	0.443
No. of non-H atoms		
Protein	1006	1011
Ion	1 Na^+^, 1 Cl	1 Na^+^, 1 Cl
Water	63	44
Total	1071	1055
R.m.s. deviations		
Bonds ()	0.008	0.007
Angles ()	1.023	0.968
Average *B* factors (^2^)		
Protein	30.1	45.2
Ion	34.1	49.8
Water	39.8	48.2
Ramachandran plot		
Most favoured (%)	98.4	96.8
Allowed (%)	1.6	3.2
