# Supplementary material for: Characterization and use of the spent beam for serial operation of LCLS
Source: J Synchrotron Radiat. 2015 Apr 11;22(Pt 3):634–43. doi: 10.1107/S1600577515004002 (PMC4416680; doi:10.1107/S1600577515004002)
Supplement: Supplementary file 1 [file s-22-00634-sup1.pdf]

JOURNAL OF SYNCHROTRON RADIATION

Volume 22 (2015)

Supporting information for article:

Characterization and use of the spent beam for serial operation of LCLS

Sébastien Boutet, Lutz Foucar, Thomas R. M. Barends, Sabine Botha, R. Bruce Doak, Jason E. Koglin, Marc Messerschmidt, Karol Nass, Ilme Schlichting, M. Marvin Seibert, Robert L. Shoeman and Garth J. Williams

S1. Wavefront Propagation Results at Best Focus

Presented in this supplementary section are the wavefront propagation results before and after optical elements along the propagation path for an energy of 6.719 keV which produces the best focus for lens stack number 1. The simulations were performed in 1D for both the horizontal and vertical axes separately. The propagation sequence is the following

Source → HOMS Mirrors → KB Mirrors → Be Lenses → Interaction Point

For the vertical direction, the HOMS mirrors are essentially infinite and this step is just bypassed since the mirrors are assumed perfect.

For the horizontal direction, the beam is propagated over the HOMS mirrors and this was done both for the case of a perfect but finite length HOMS as well as for a case with a figure error that generates a two lobe beam profile at the CXI location as observed in reality. This figure error was simulated as a simple triangular shaped phase gradient which leads to splitting of the beam in two main lobes. For the case of simulated HOMS figure error, the lenses were then moved off center of the beam profile to be instead centered on one of the two lobes of the beam, as was done in real life as shown on Fig. 2 in the main article. This shift of the lenses causes the peak in the beam profile at the interaction region to be shifted from the center of the simulation, as expected.

The source was simulated as a 1.5 mJ source and losses due to both transmission and aperturing were tracked during the simulation. Since the simulation were 1D only the transmission losses need to be counted for each direction, which means in practice that for the case of the VFM starting with 1.5mJ at the source and ending with 0.27 mJ at the interaction point and for the HFM ending with 0.47 mJ, the total transmission would then be 0.27 x 0.47/(1.5x1.5)= 0.056 or 5.6%.. Another factor of $\pi/4$ is required due to the circular aperture of the lenses instead of square. Yielding a 4.4% efficiency for the entire optical path. The efficiency is larger in the horizontal direction because the clear aperture of the VFM is significantly smaller due to its much smaller average incidence angle cause by the high curvature required by the shorter focal length.

The simulated efficiency of the refocusing part alone is higher, when removing the upstream losses from the calculation. One then gets 7.7% simulated efficiency for the refocusing at 6.7 keV. With Be transmission losses smaller at higher energies, this should represent a lower bound for the efficiency of the system studied here.

S1.1. VFM

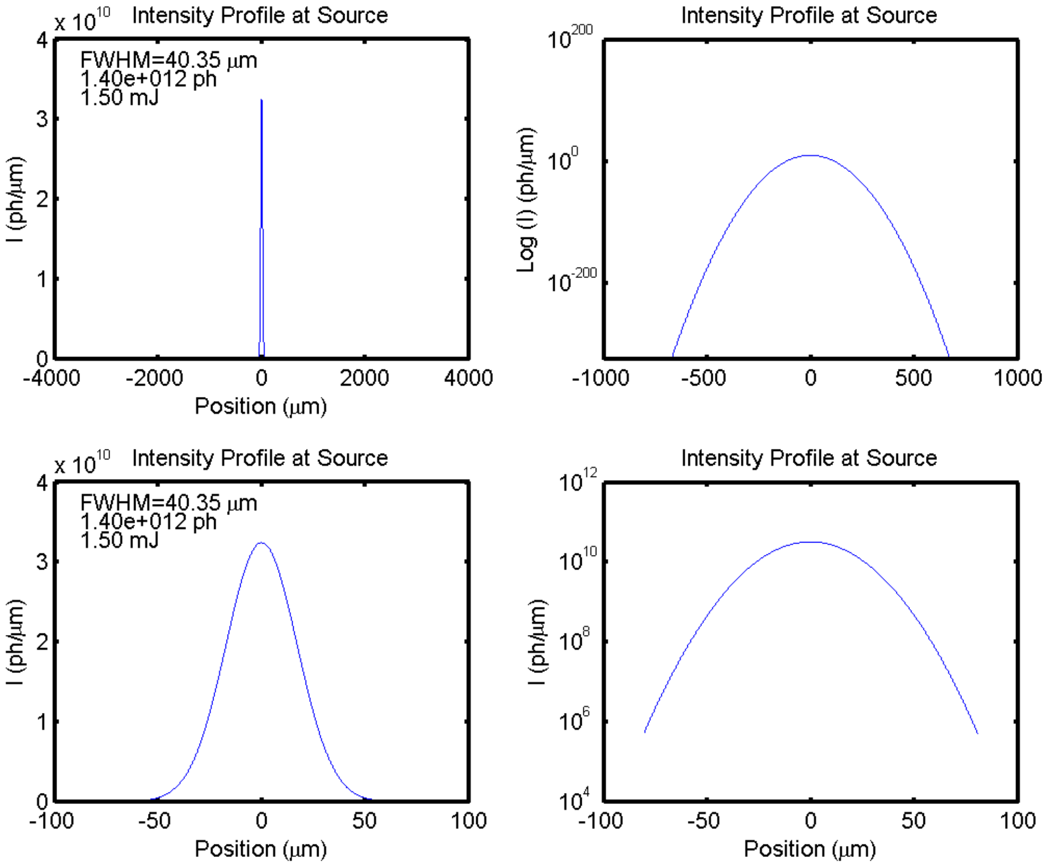

Figure S1 Beam profile at the source in the vertical direction for 6.719 keV.

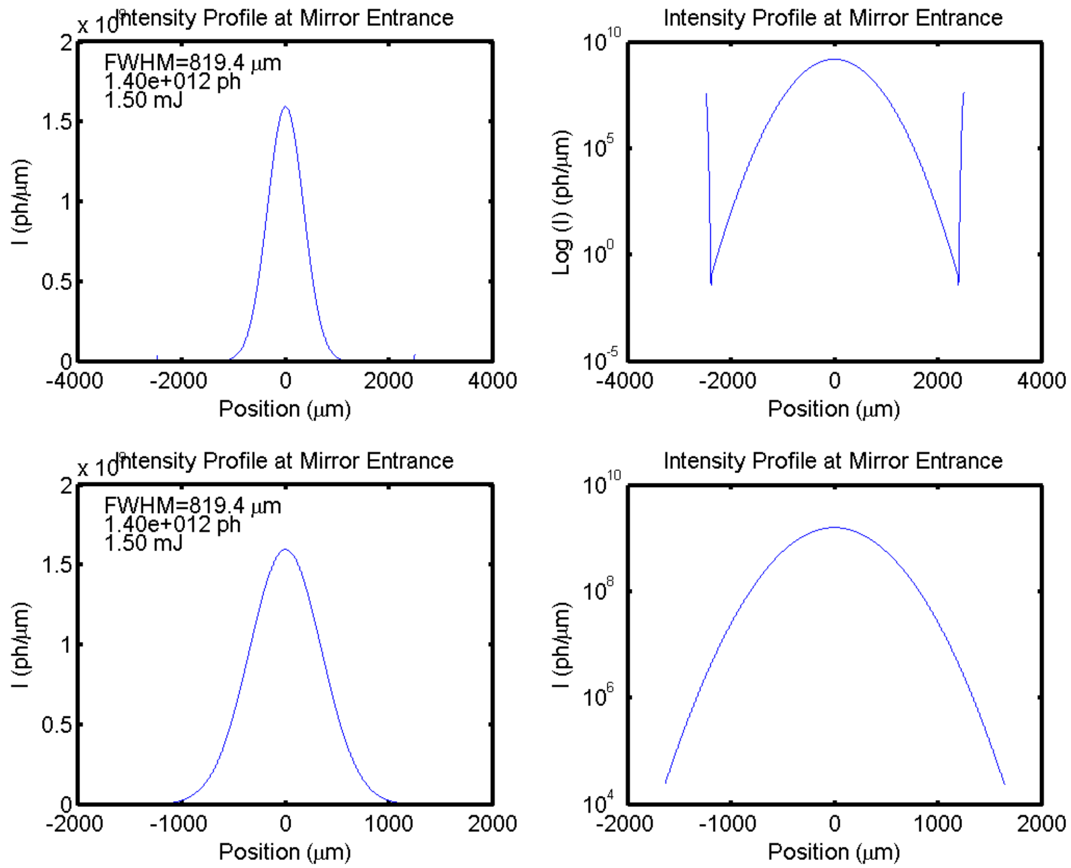

Figure S2 Beam profile at the VMF entrance in the vertical direction for 6.719 keV.

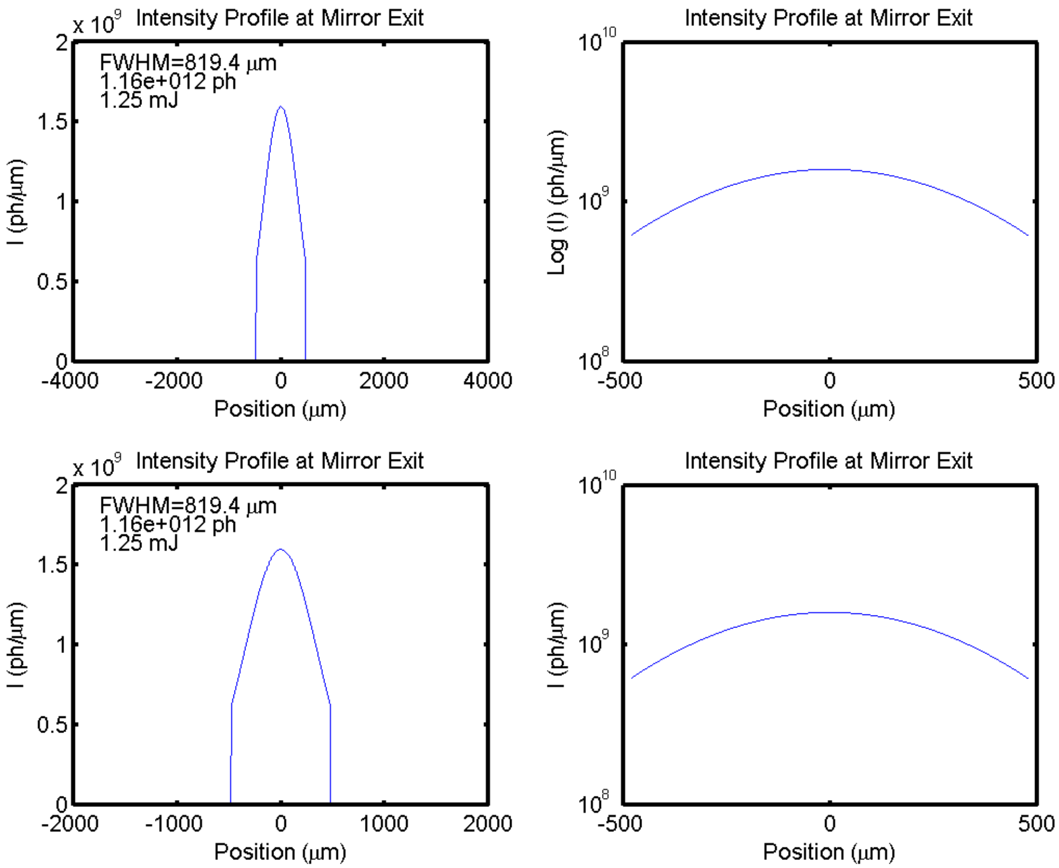

Figure S3 Beam profile at the VFM exit in the vertical direction for 6.719 keV.

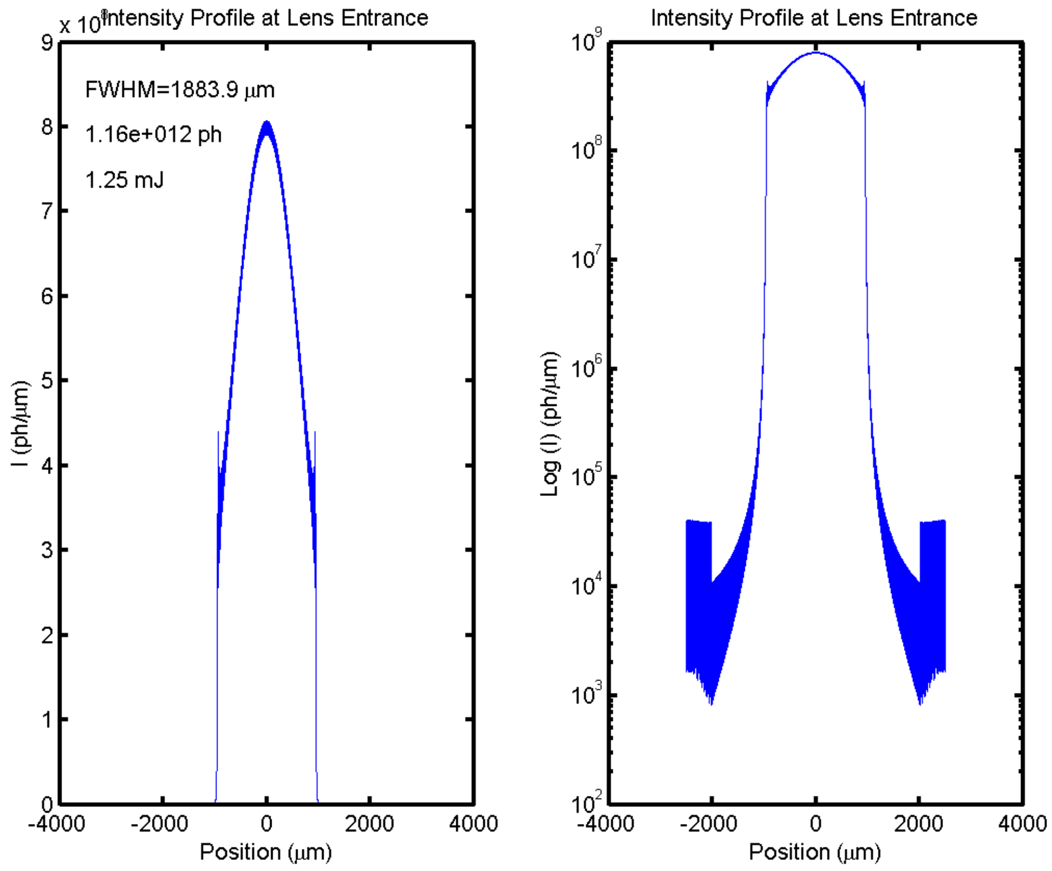

Figure S4 Beam profile at the Be lens entrance in the vertical direction for 6.719 keV.

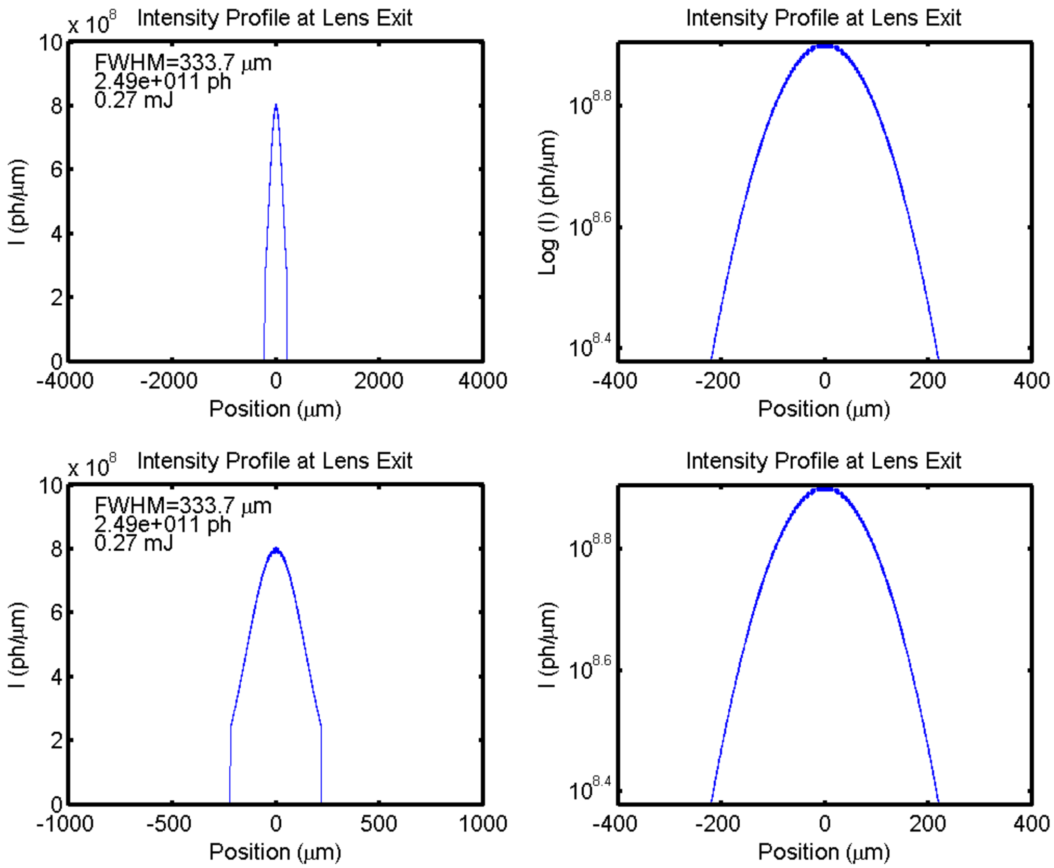

Figure S5 Beam profile at the Be lens exit in the vertical direction for 6.719 keV.

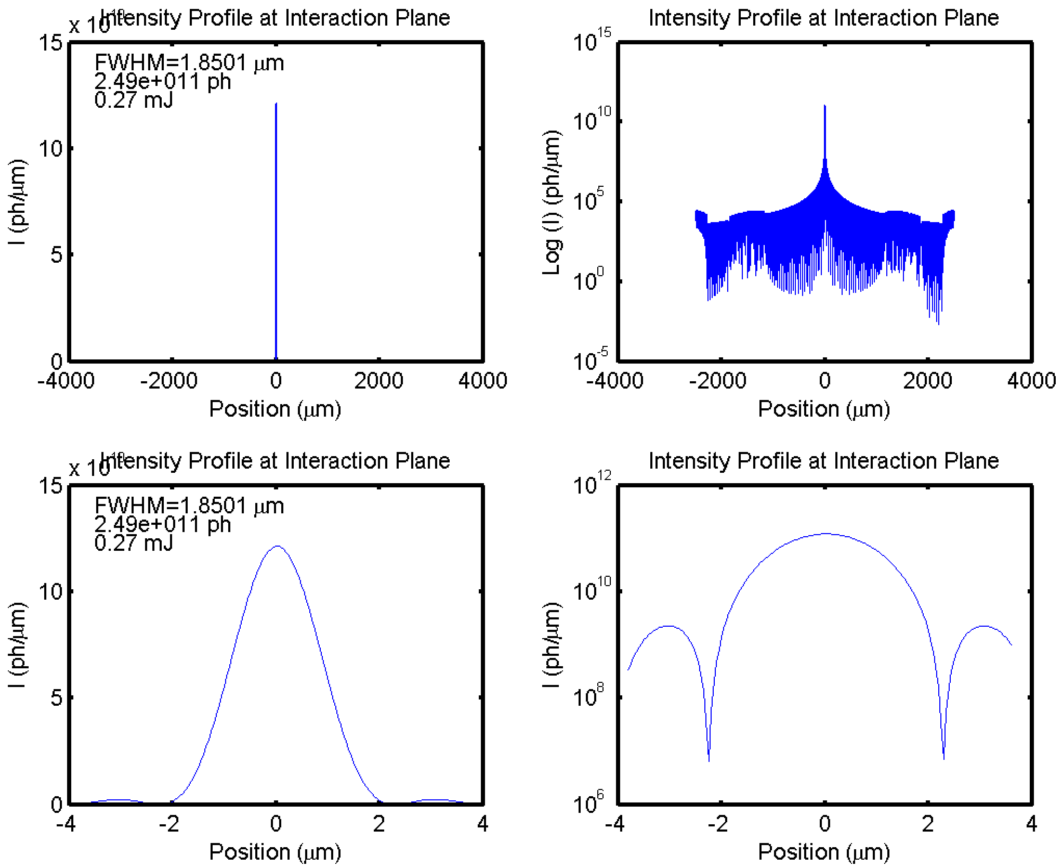

Figure S6 Beam profile at the refocused interaction plane in the vertical direction for 6.719 keV.

S1.2. HFM with no HOMS Figure error

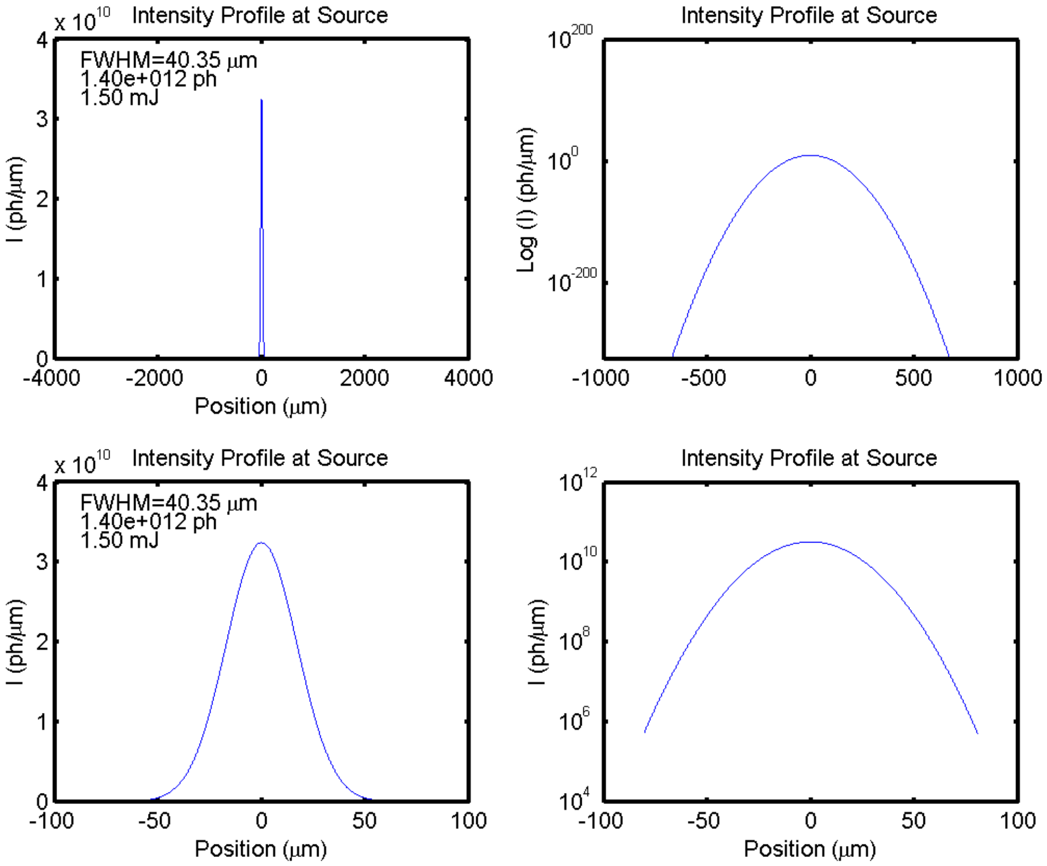

Figure S7 Beam profile at the source in the horizontal direction for 6.719 keV.

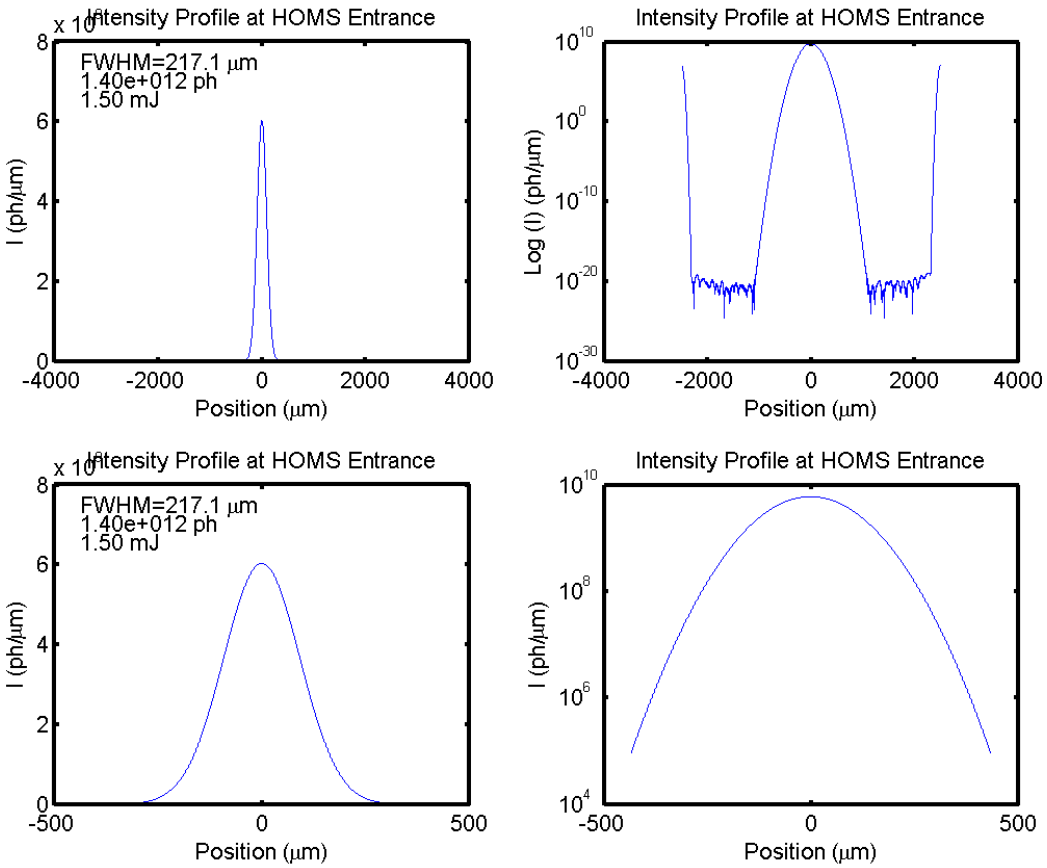

Figure S8 Beam profile at the HOMS entrance in the horizontal direction for 6.719 keV.

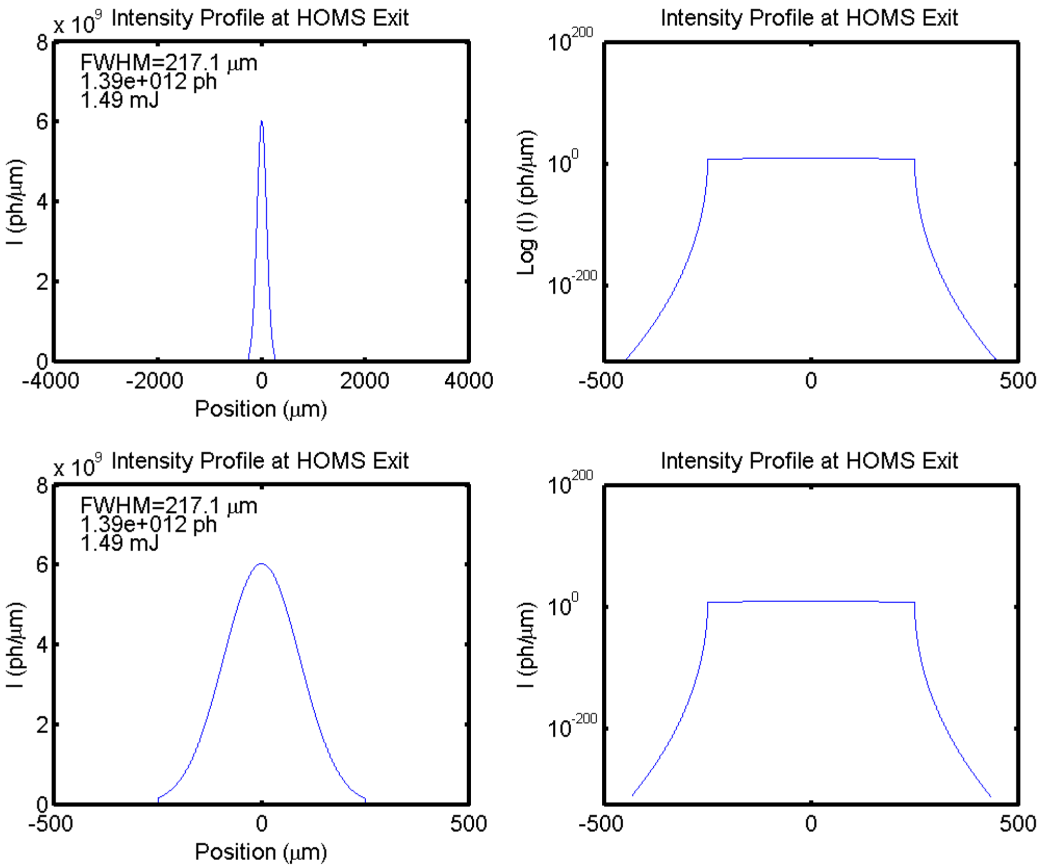

Figure S9 Beam profile at the HOMS exit in the horizontal direction for 6.719 keV.

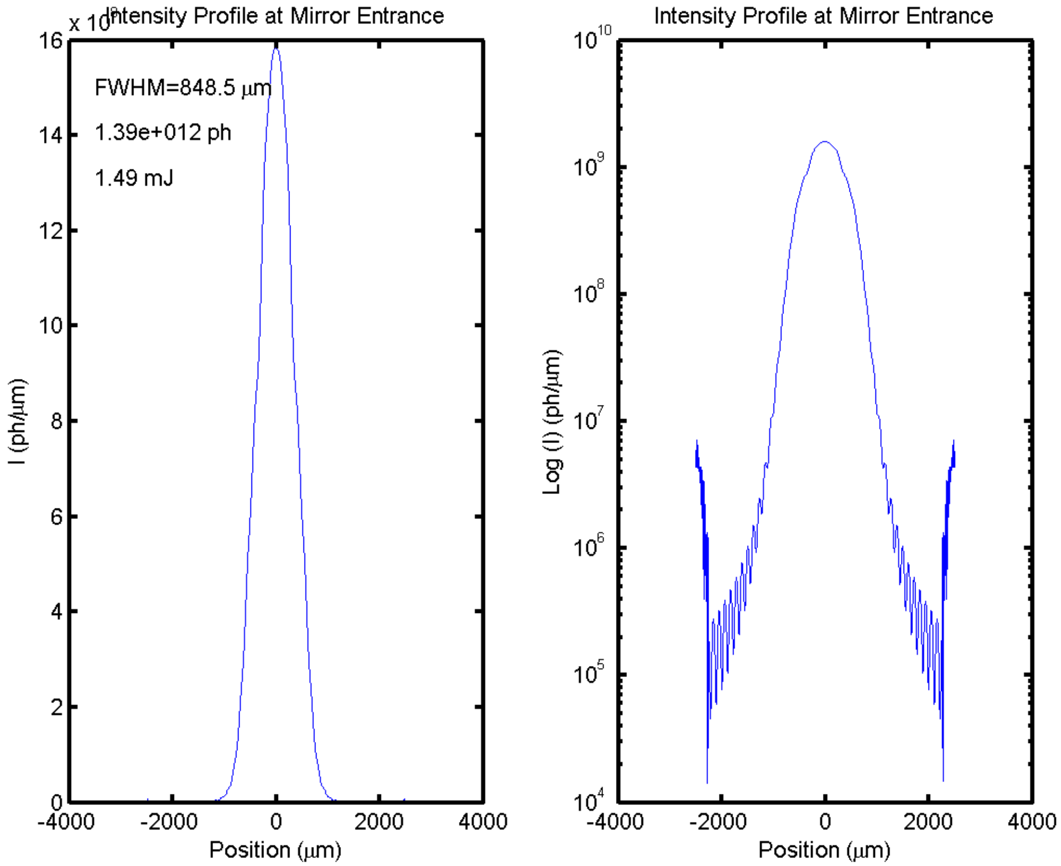

Figure S10 Beam profile at the HFM entrance in the horizontal direction for 6.719 keV.

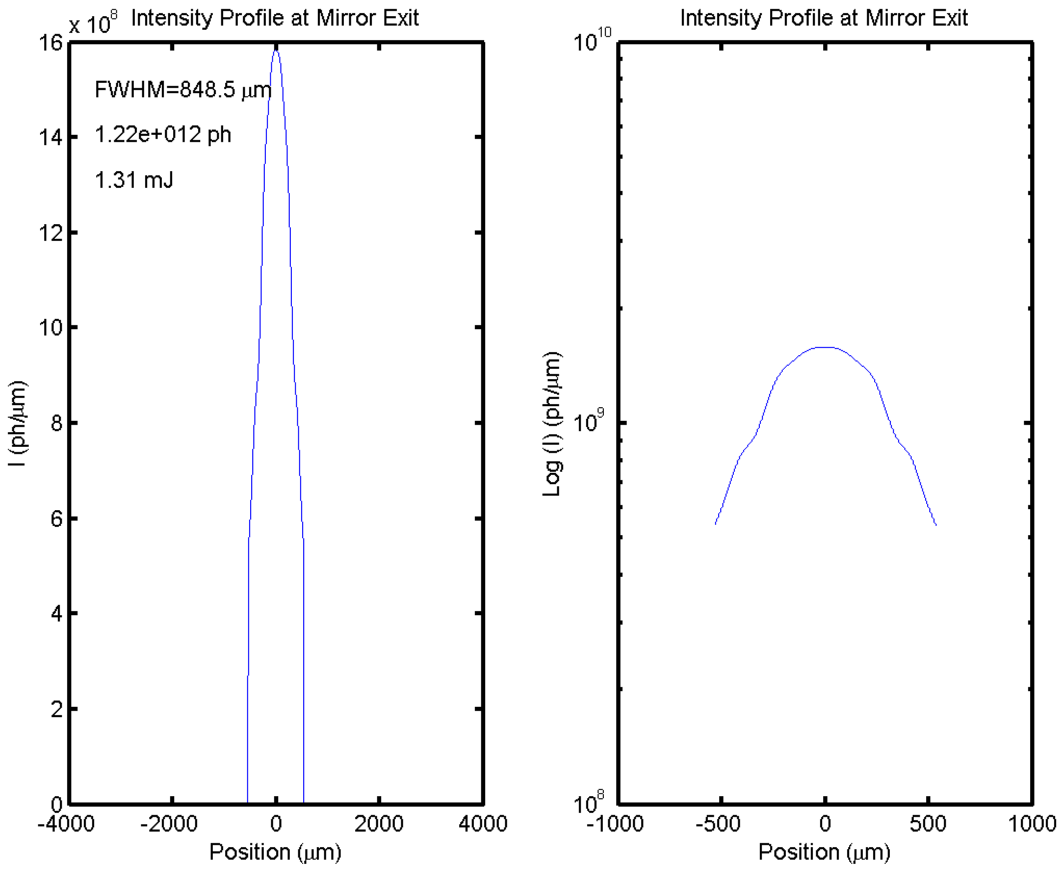

Figure S11 Beam profile at the HFM exit in the horizontal direction for 6.719 keV.

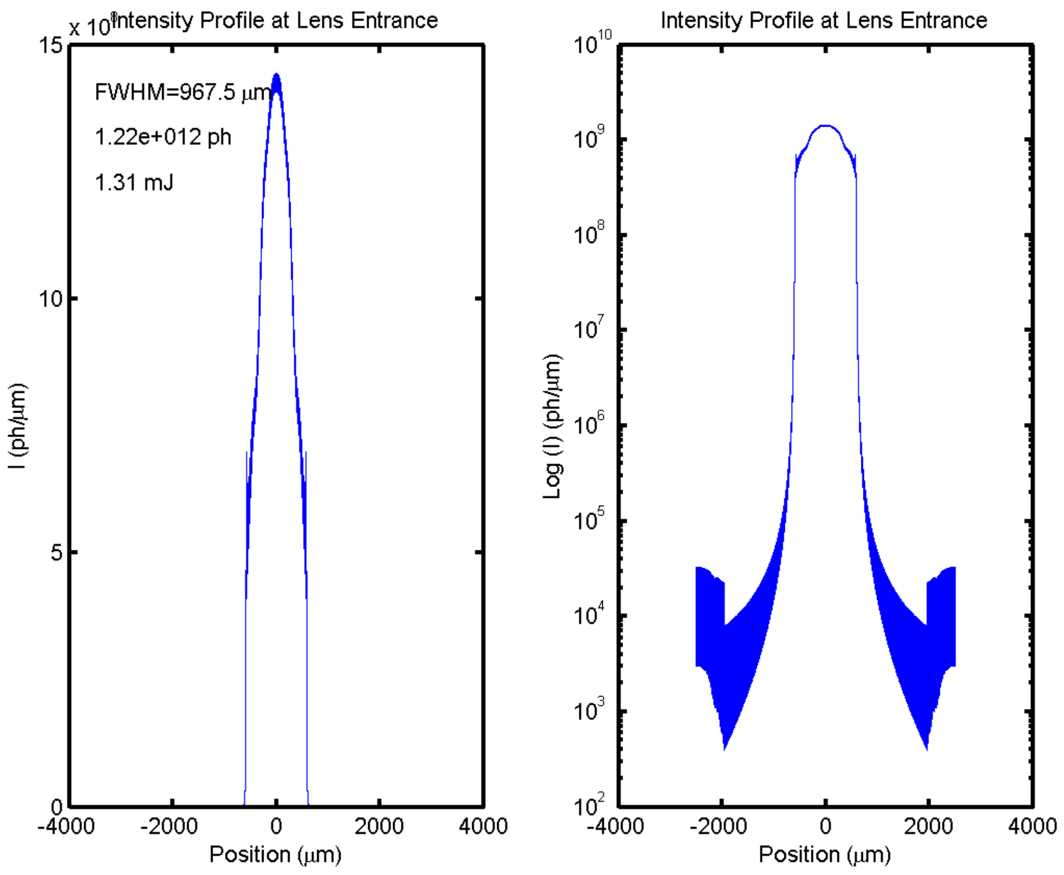

Figure S12 Beam profile at the Be lens entrance in the horizontal direction for 6.719 keV.

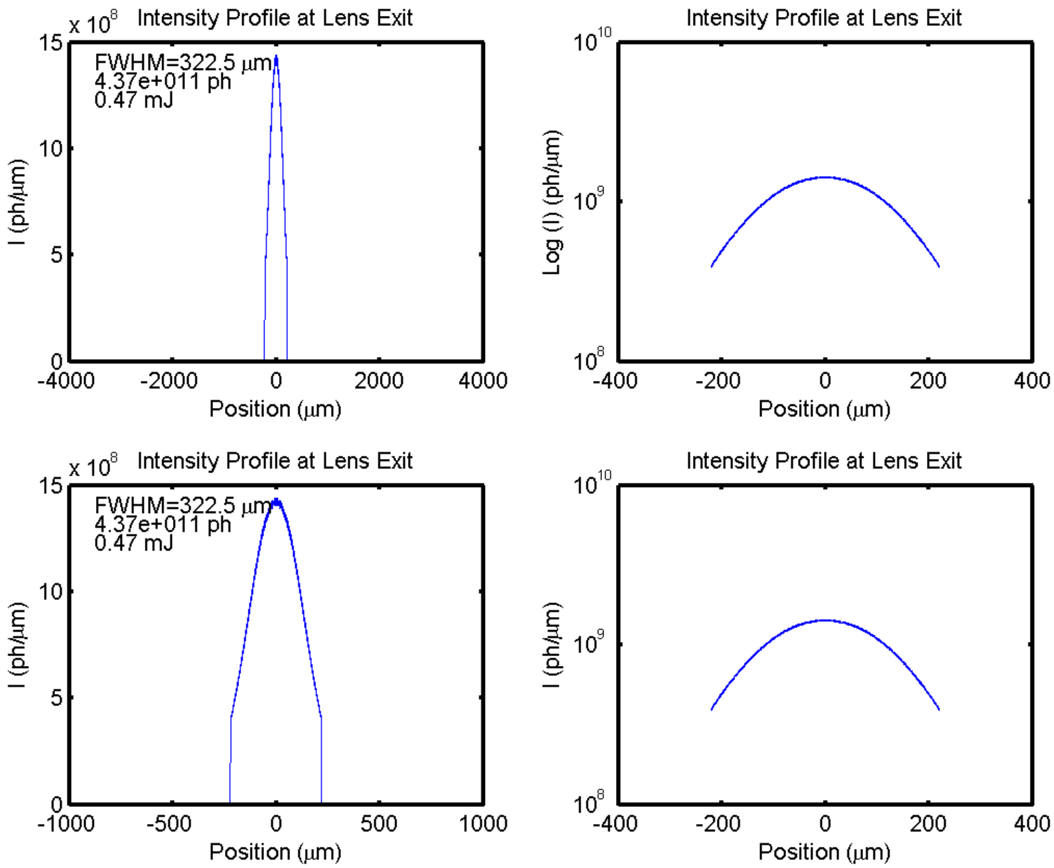

Figure S13 Beam profile at the Be lens exit in the horizontal direction for 6.719 keV.

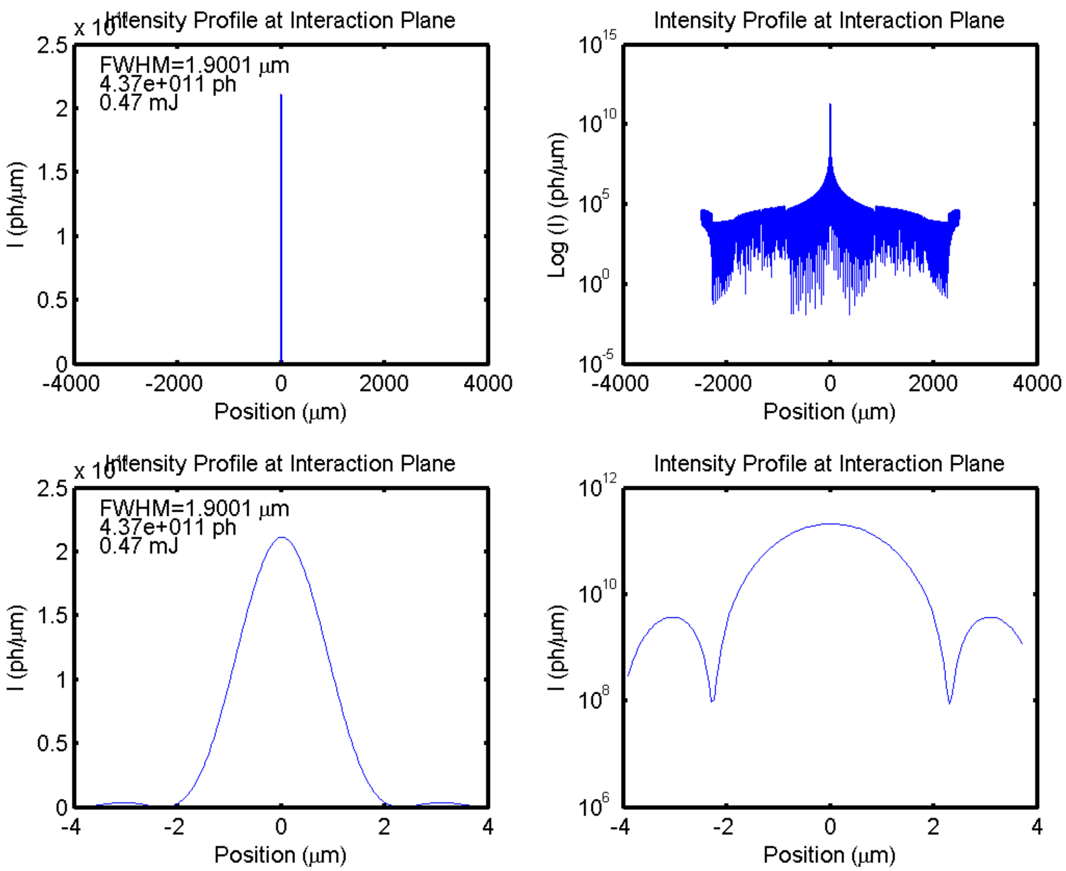

Figure S14 Beam profile at the refocused interaction plane in the horizontal direction for 6.719 keV.

S1.3. HFM with simple simulated HOMS Figure error

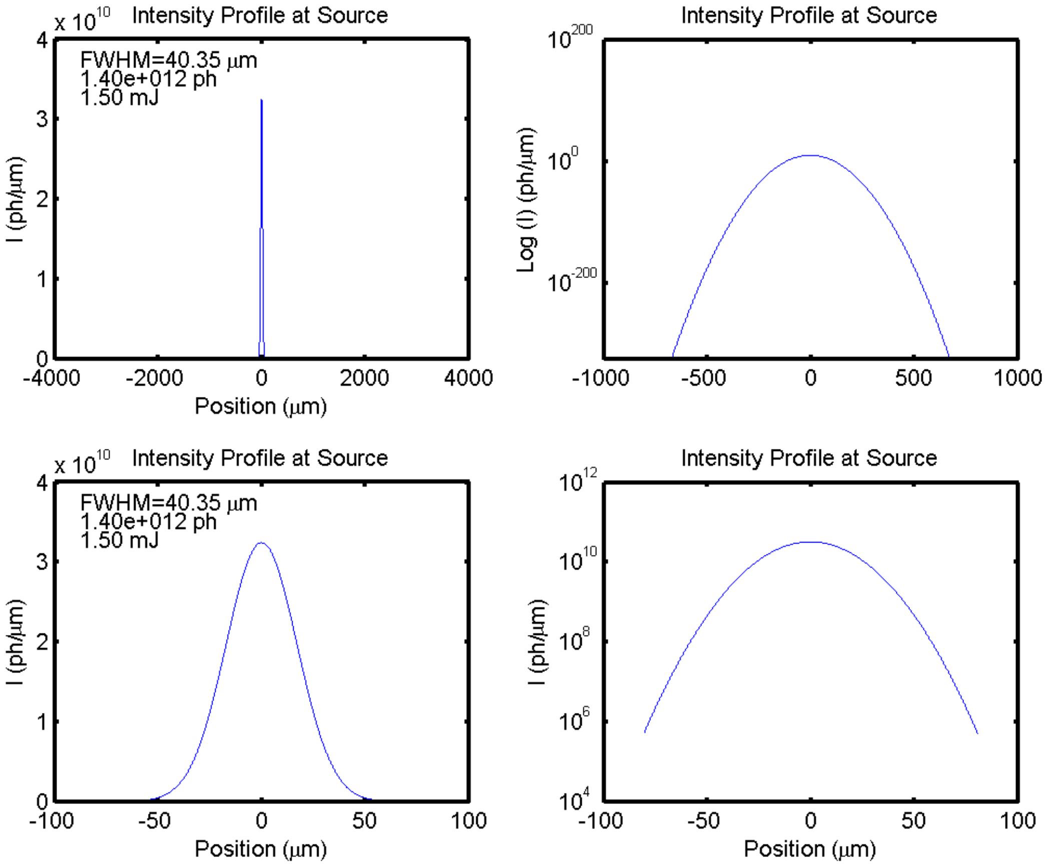

Figure S15 Beam profile at the source in the horizontal direction for 6.719 keV.

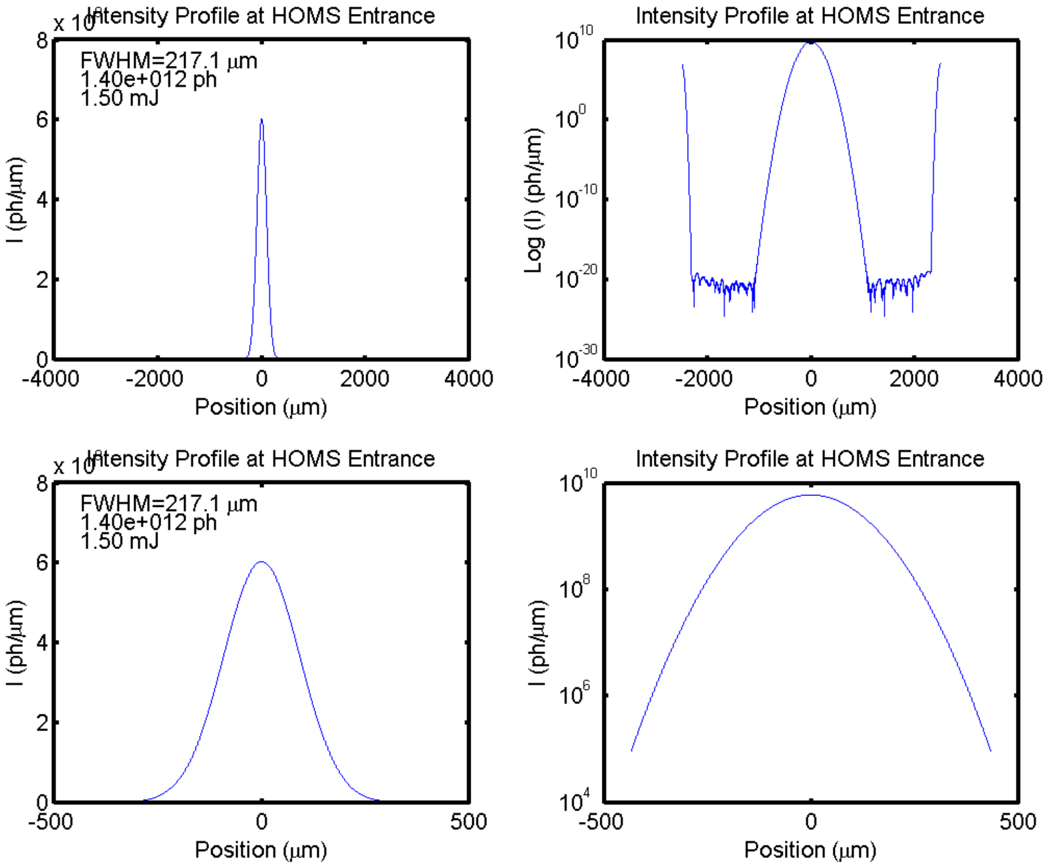

Figure S16 Beam profile at the HOMS entrance in the horizontal direction for 6.719 keV.

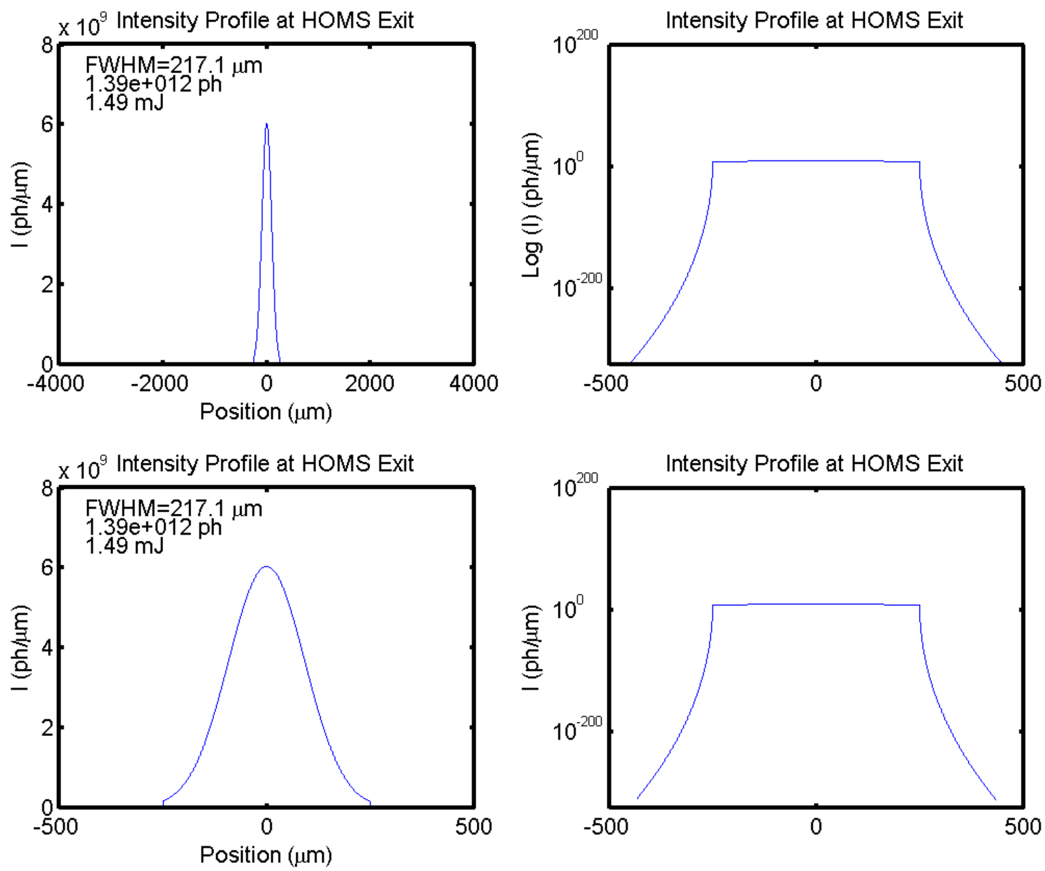

Figure S17 Beam profile at the HOMS exit in the horizontal direction for 6.719 keV.

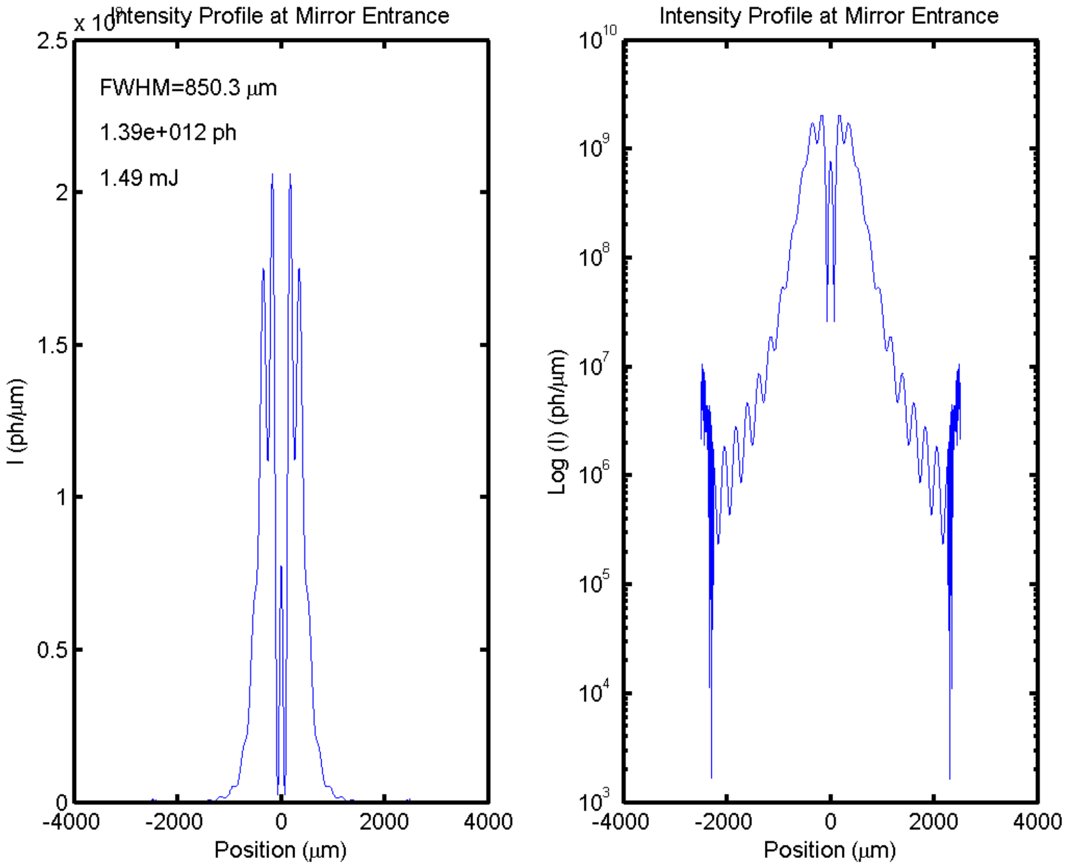

Figure S18 Beam profile at the HFM entrance in the horizontal direction for 6.719 keV.

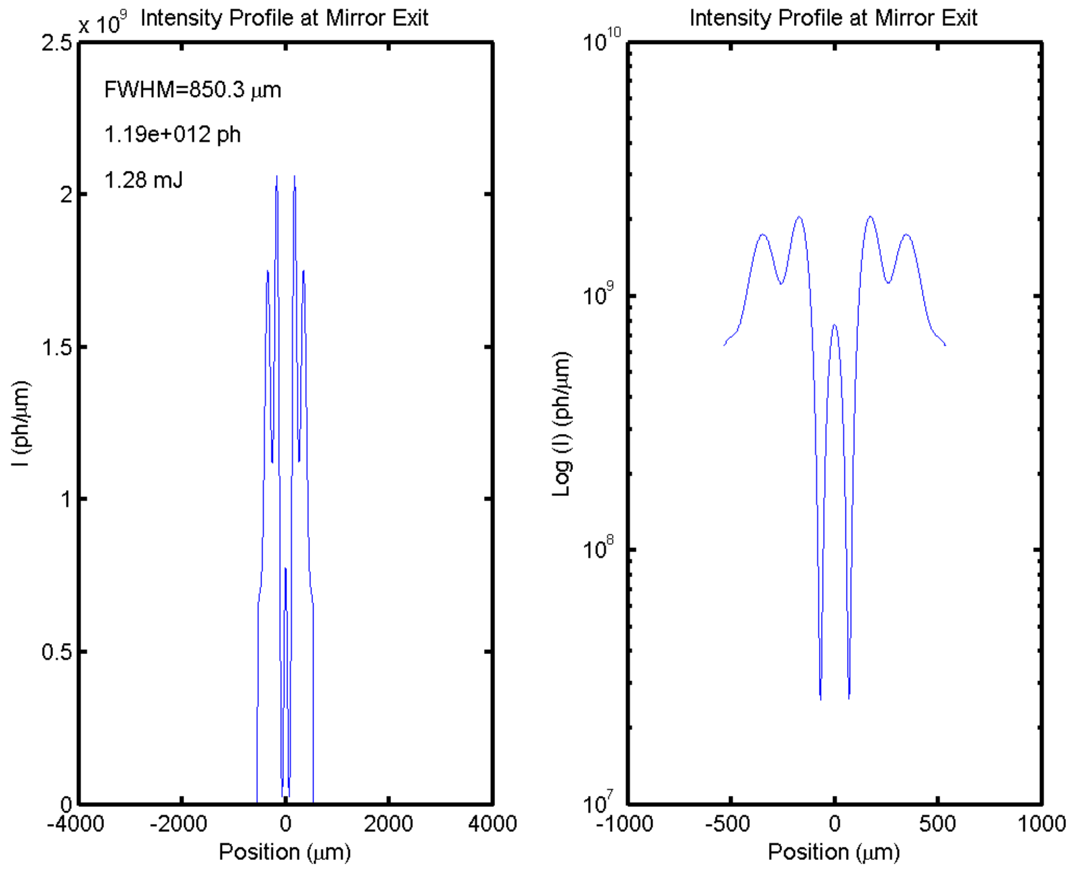

Figure S19 Beam profile at the HFM exit in the horizontal direction for 6.719 keV.

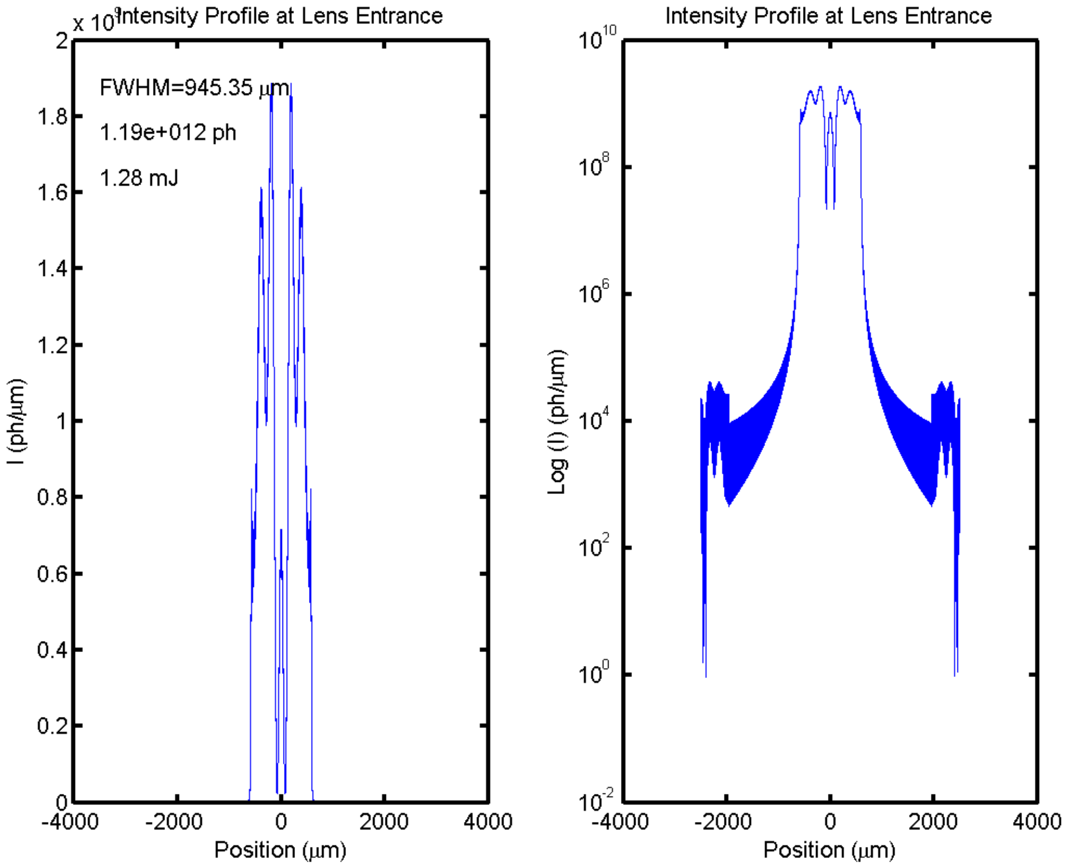

Figure S20 Beam profile at the Be lens entrance in the horizontal direction for 6.719 keV.

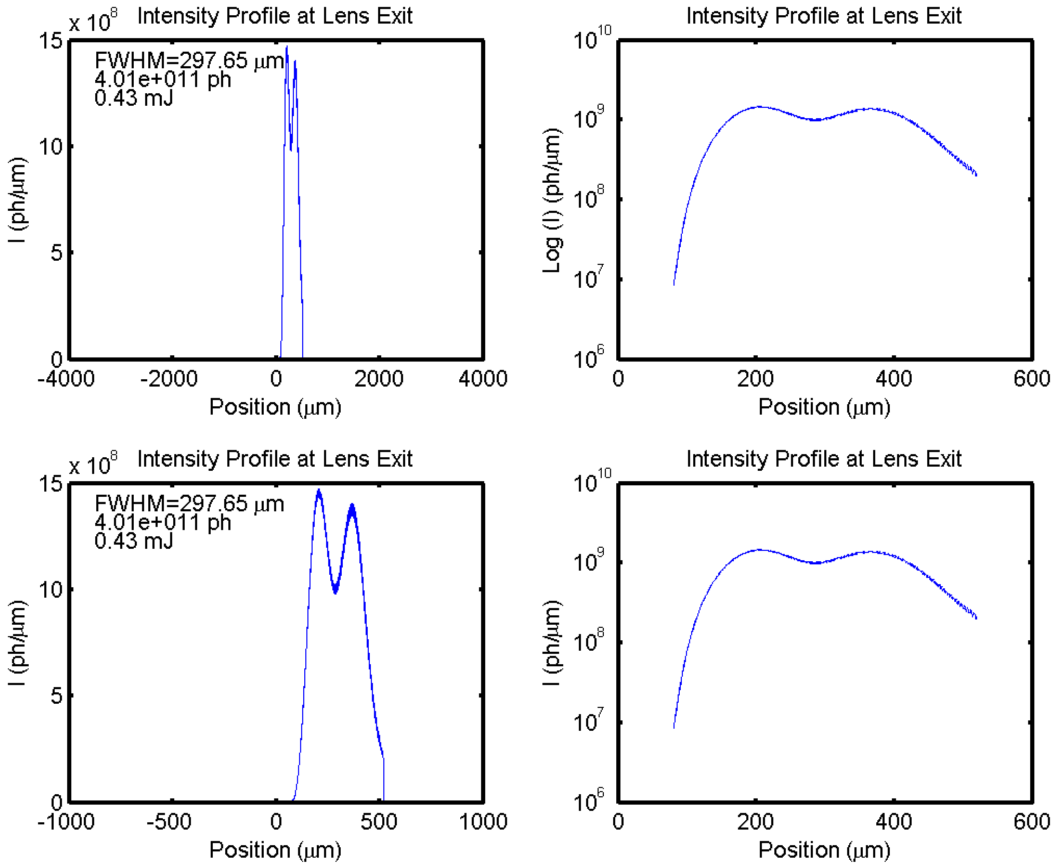

Figure S21 Beam profile at the Be lens exit in the horizontal direction for 6.719 keV.

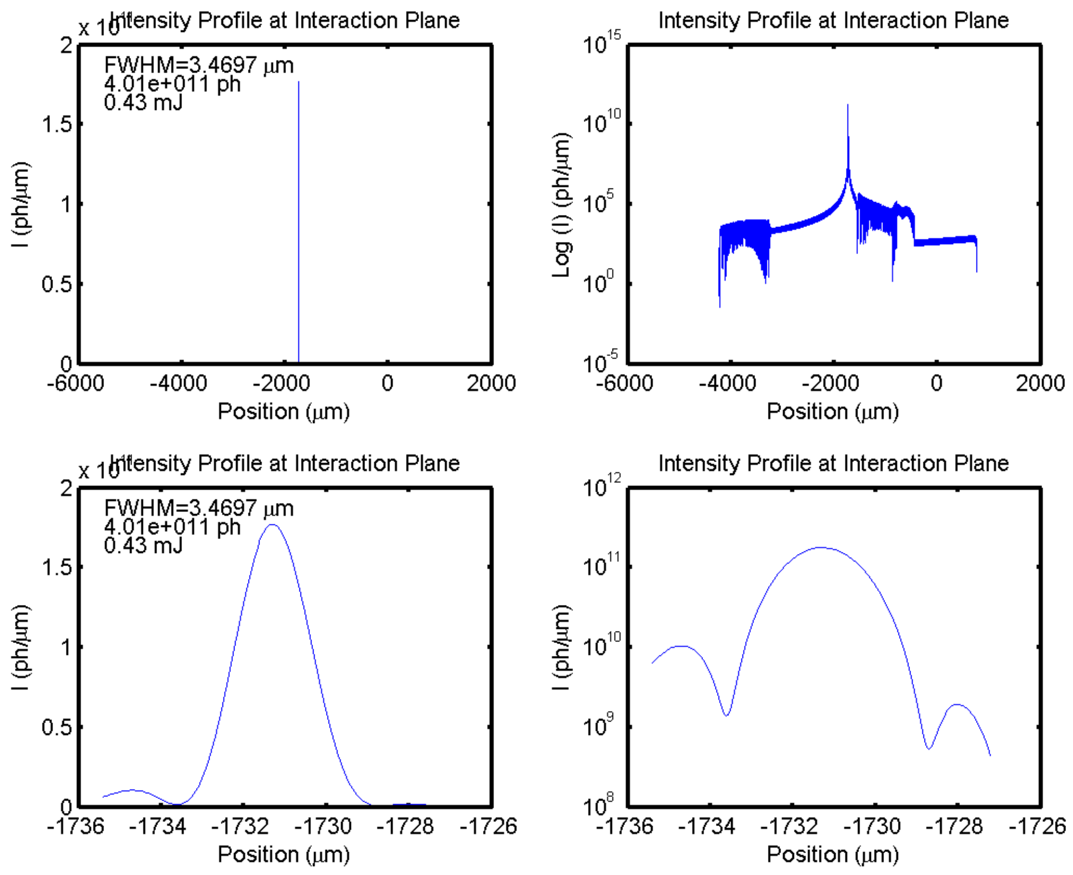

Figure S22 Beam profile at the refocused interaction plane in the horizontal direction for 6.719 keV.

S2. Wavefront Propagation Results 0.2% away from Best Focus in Photon Energy

Presented in this supplementary section are the wavefront propagation results before and after optical elements along the propagation path for an energy of 6.733 keV, which is 0.2% away from the energy that produces the best focus for lens stack number 1. The simulations were performed in 1D for both the horizontal and vertical axes separately. The propagation was done in the exact same way of for the energy producing the best focus.

Compared to the beam at an energy causing the best focus from supplementary section S1, the beam at the interaction plane is larger since it has not quite reached a focus yet.

S2.1. VFM

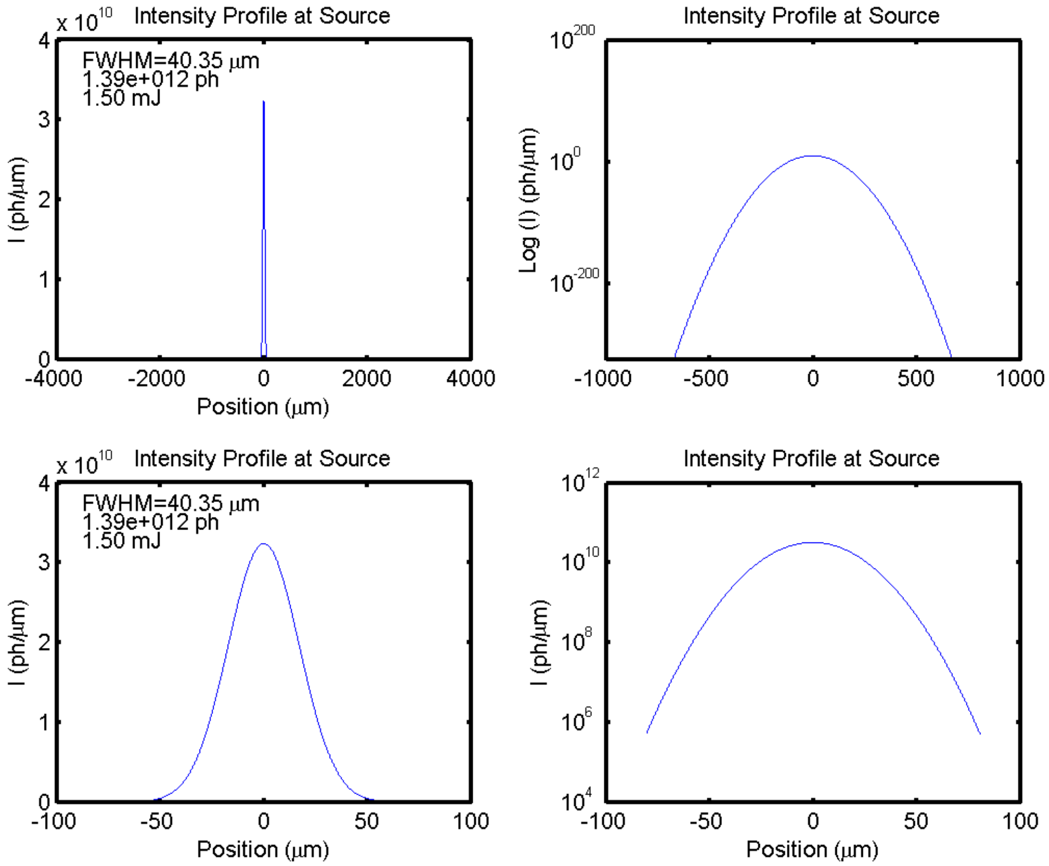

Figure S23 Beam profile at the source in the vertical direction for 6.733 keV.

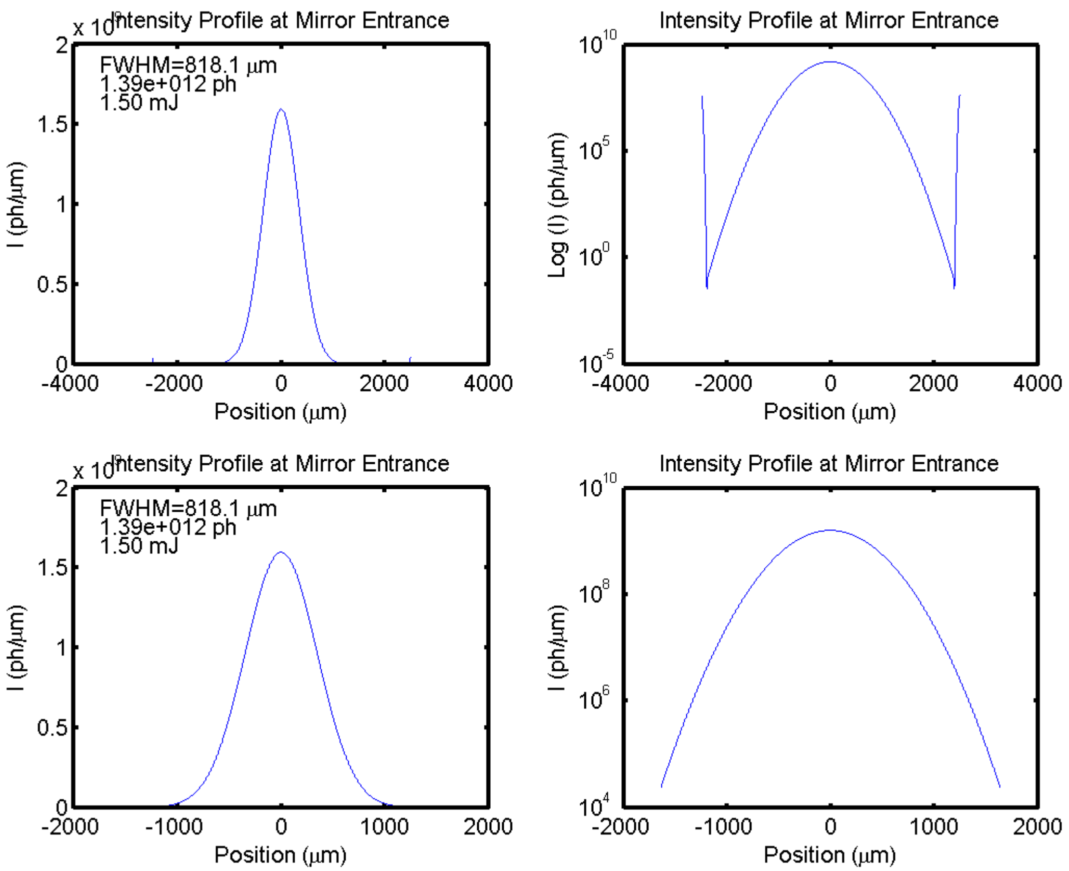

Figure S24 Beam profile at the VMF entrance in the vertical direction for 6.733 keV.

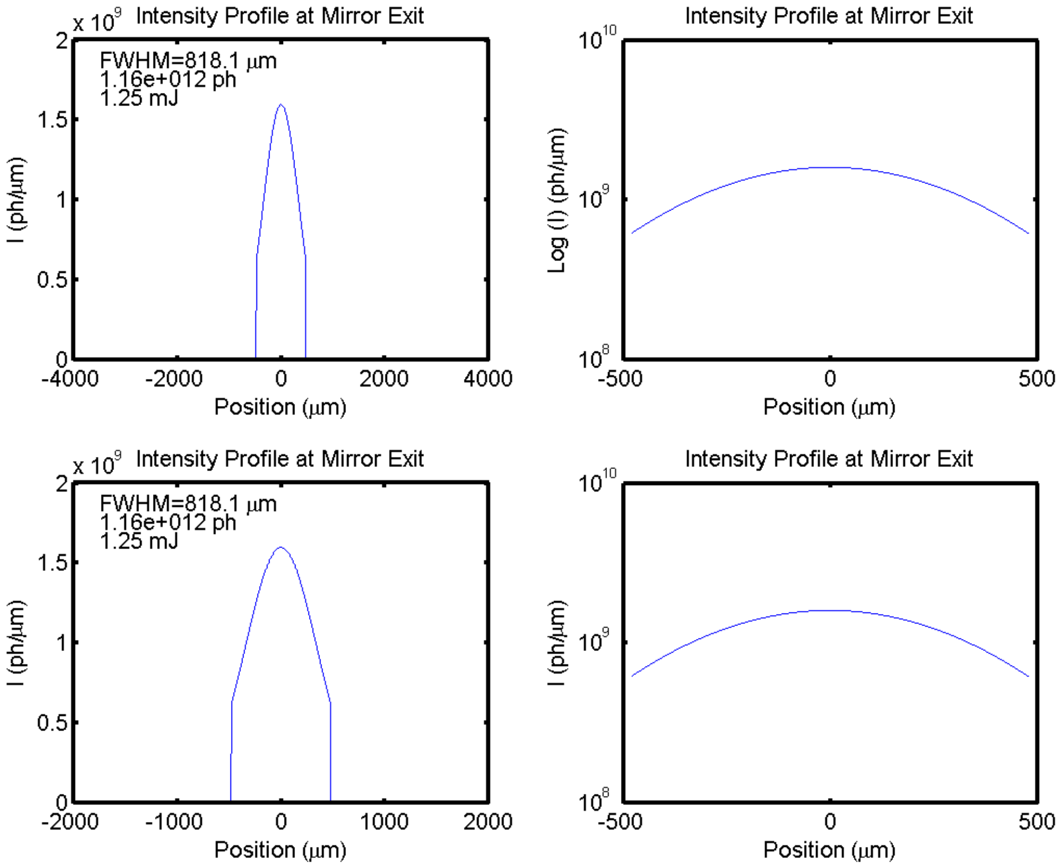

Figure S25 Beam profile at the VFM exit in the vertical direction for 6.733 keV.

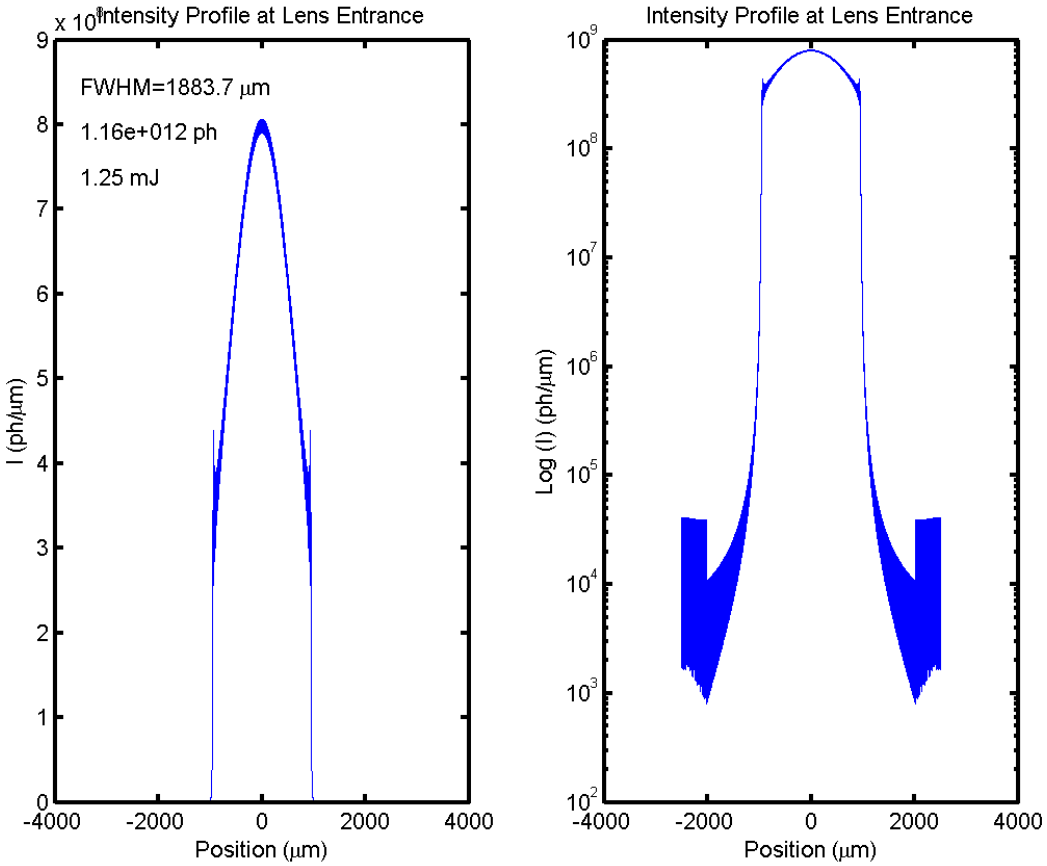

Figure S26 Beam profile at the Be lens entrance in the vertical direction for 6.733 keV.

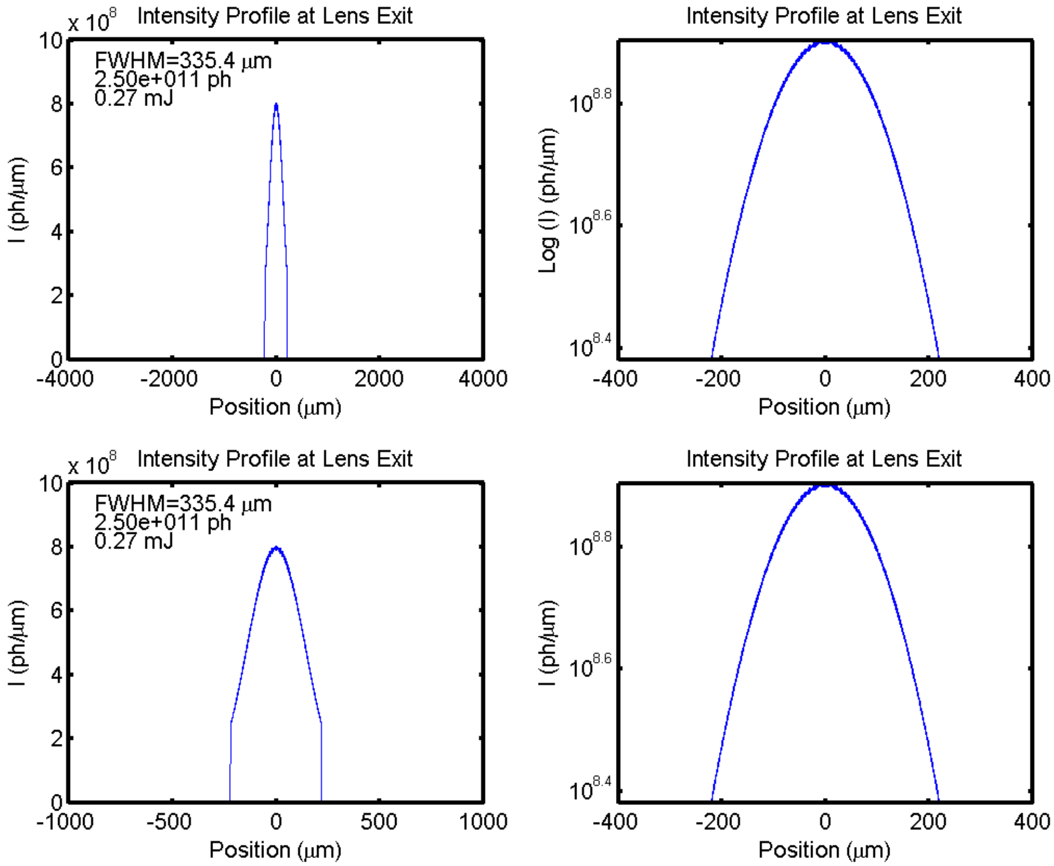

Figure S27 Beam profile at the Be lens exit in the vertical direction for 6.733 keV.

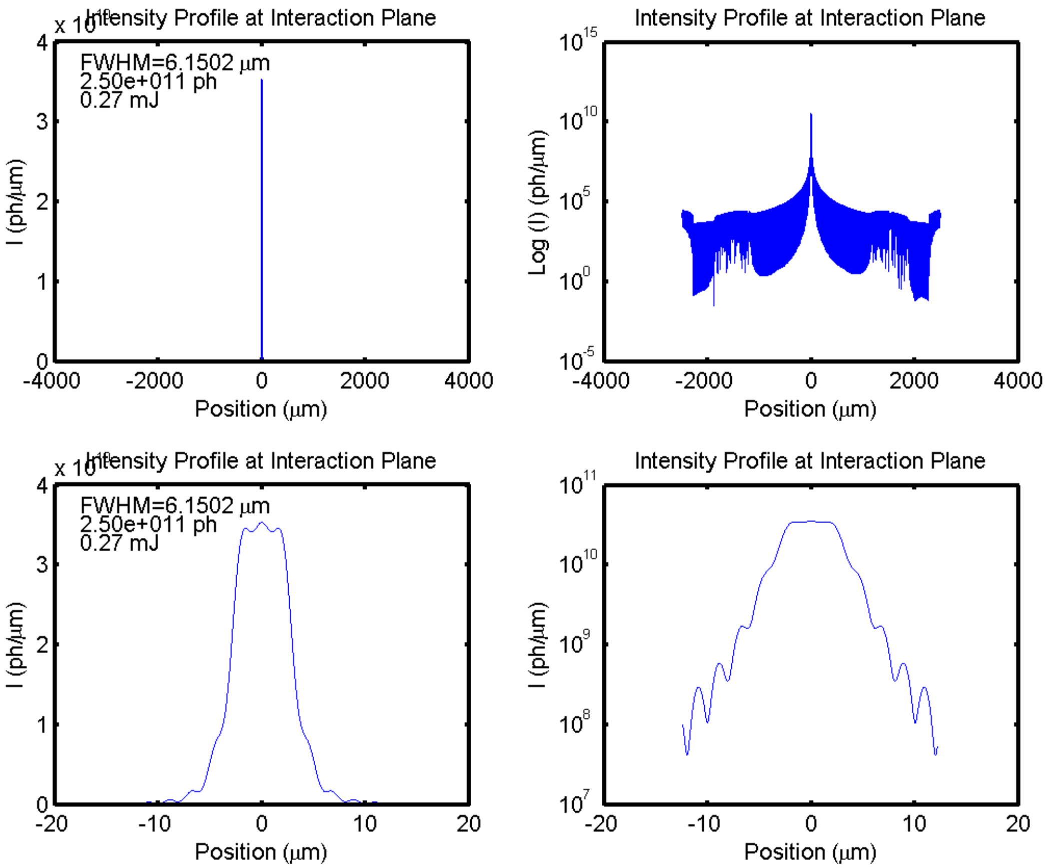

Figure S28 Beam profile at the refocused interaction plane in the vertical direction for 6.733 keV.

S2.2. HFM with no HOMS figure error

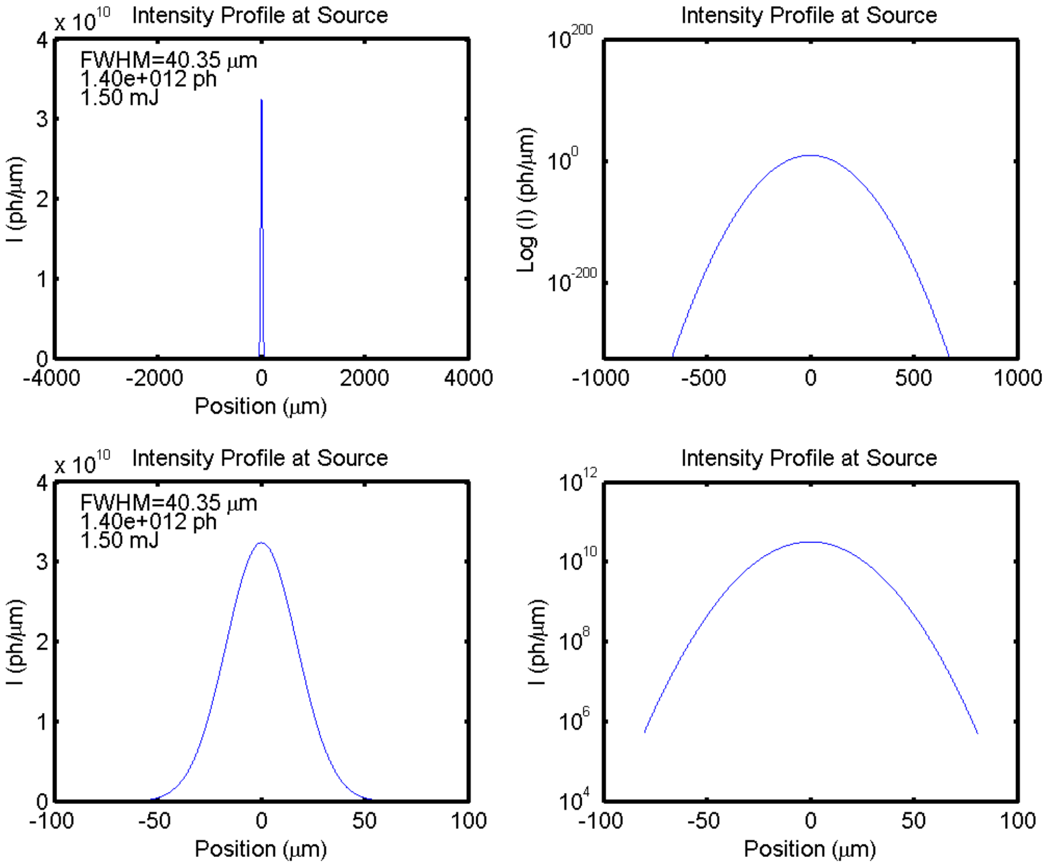

Figure S29 Beam profile at the source in the horizontal direction for 6.733 keV.

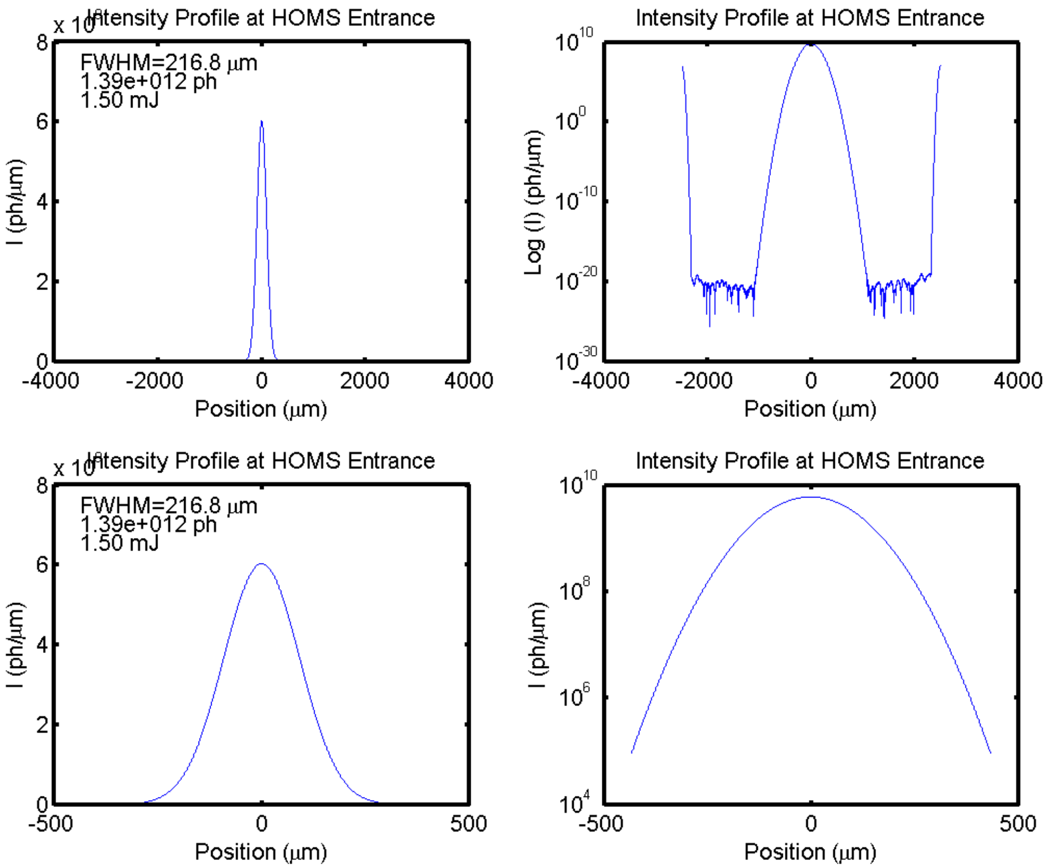

Figure S30 Beam profile at the HOMS entrance in the horizontal direction for 6.733 keV.

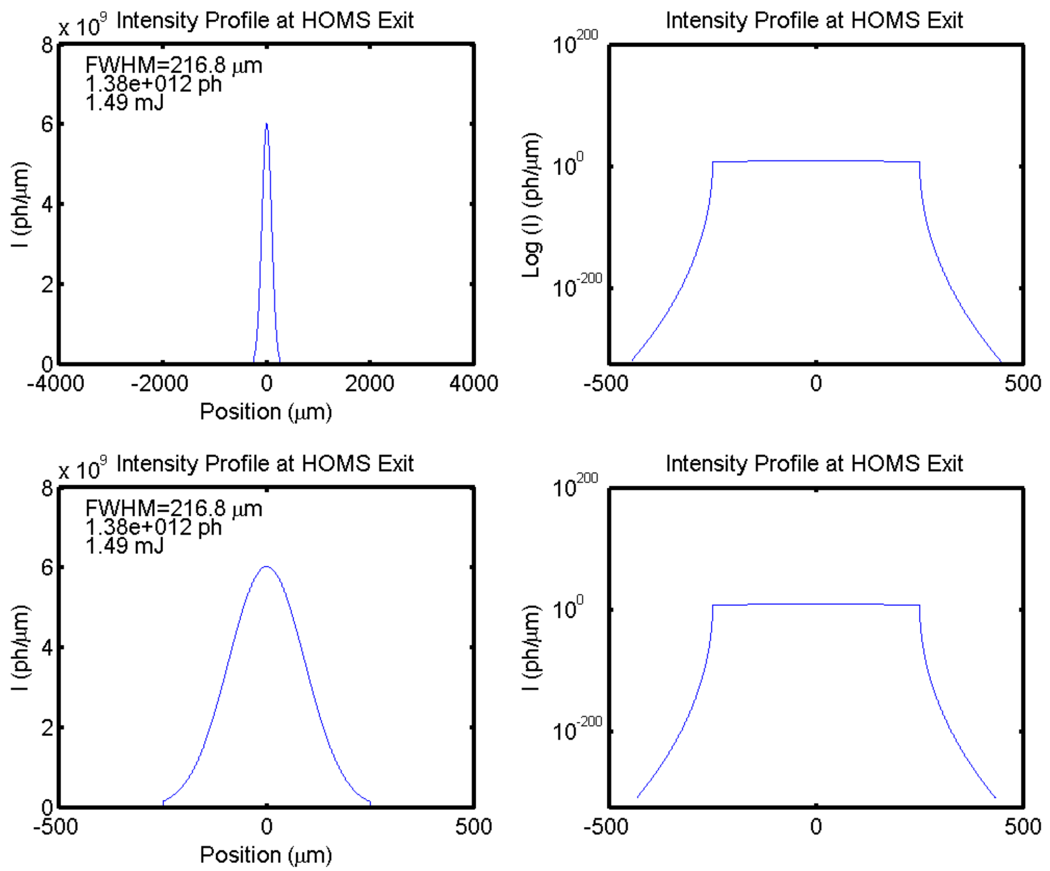

Figure S31 Beam profile at the HOMS exit in the horizontal direction for 6.733 keV.

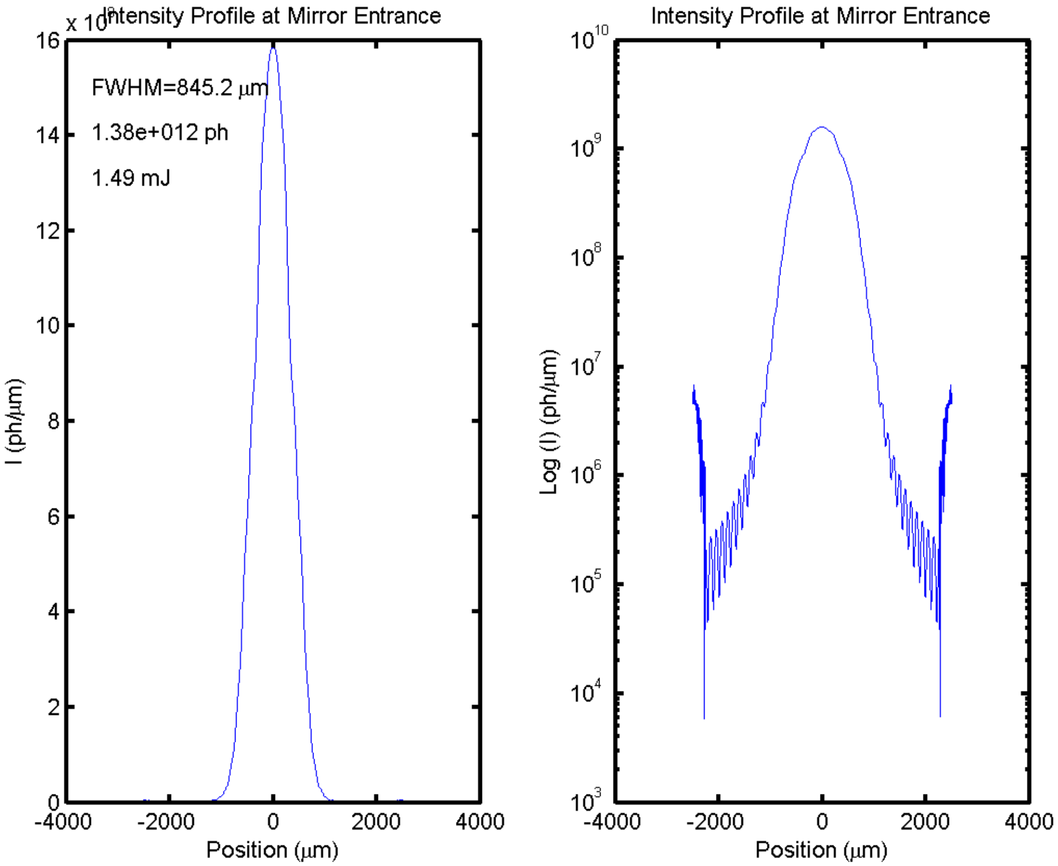

Figure S32 Beam profile at the HFM entrance in the horizontal direction for 6.733 keV.

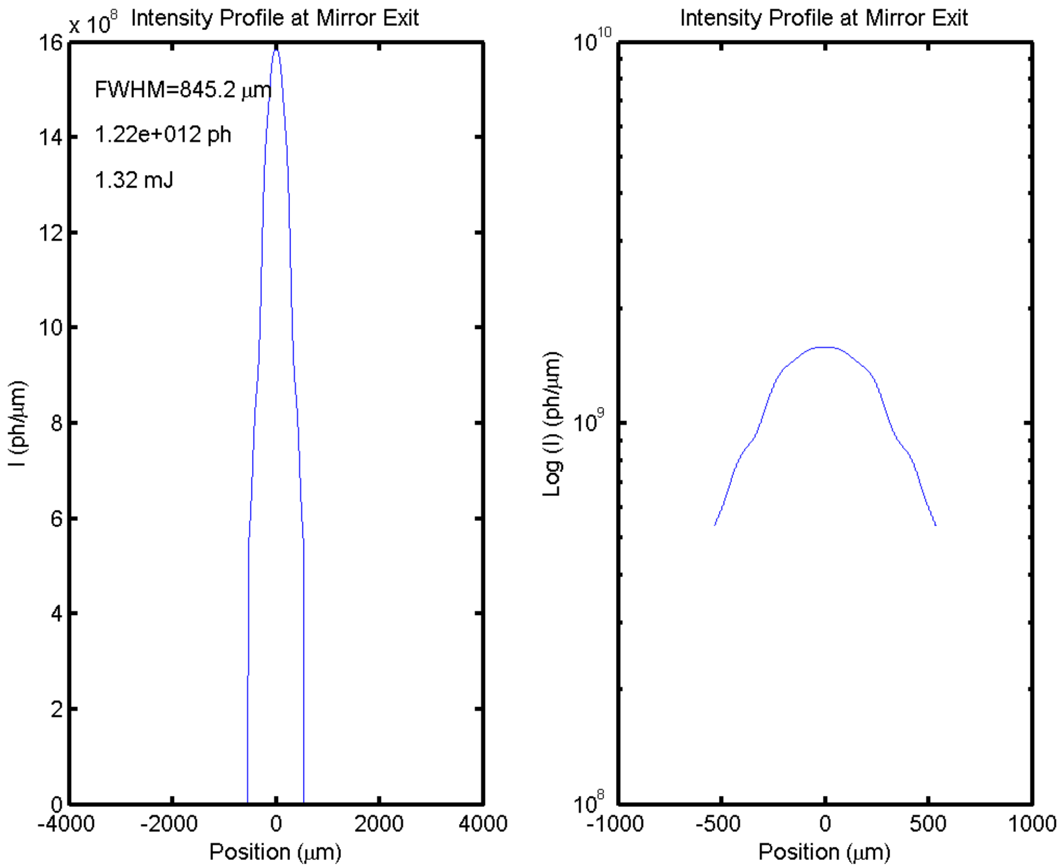

Figure S33 Beam profile at the HFM exit in the horizontal direction for 6.733 keV.

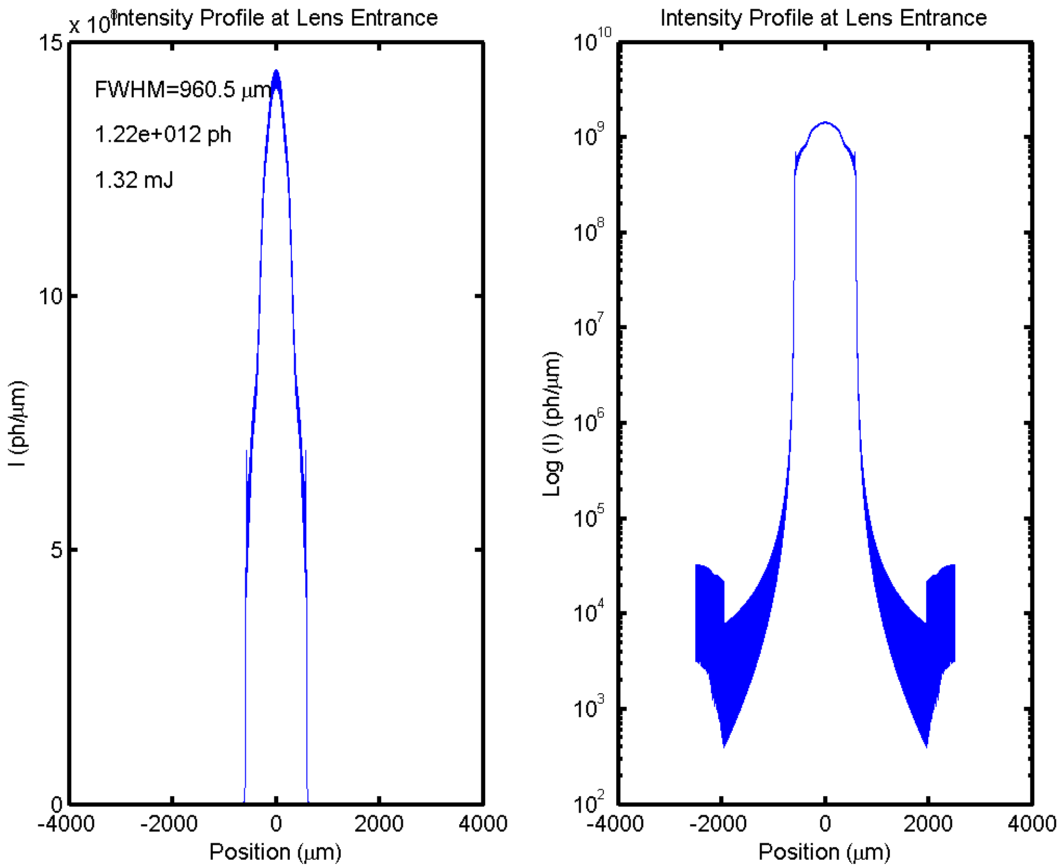

Figure S34 Beam profile at the Be lens entrance in the horizontal direction for 6.733 keV.

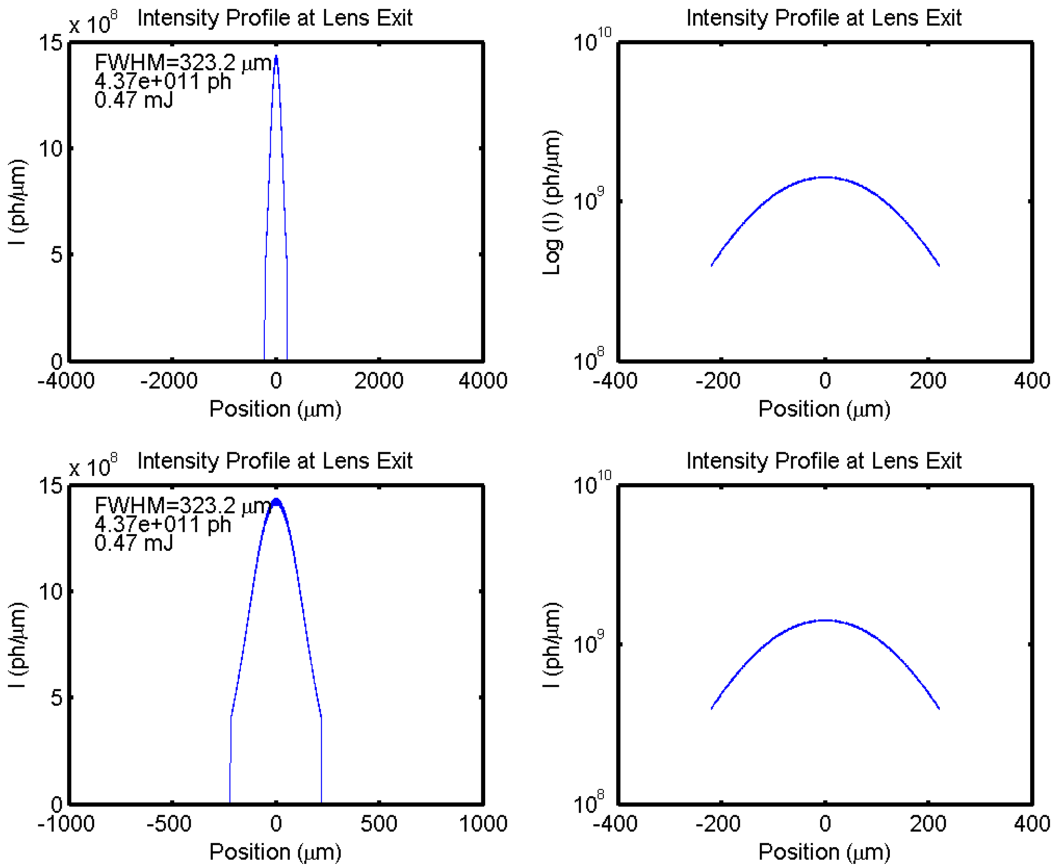

Figure S35 Beam profile at the Be lens exit in the horizontal direction for 6.733 keV.

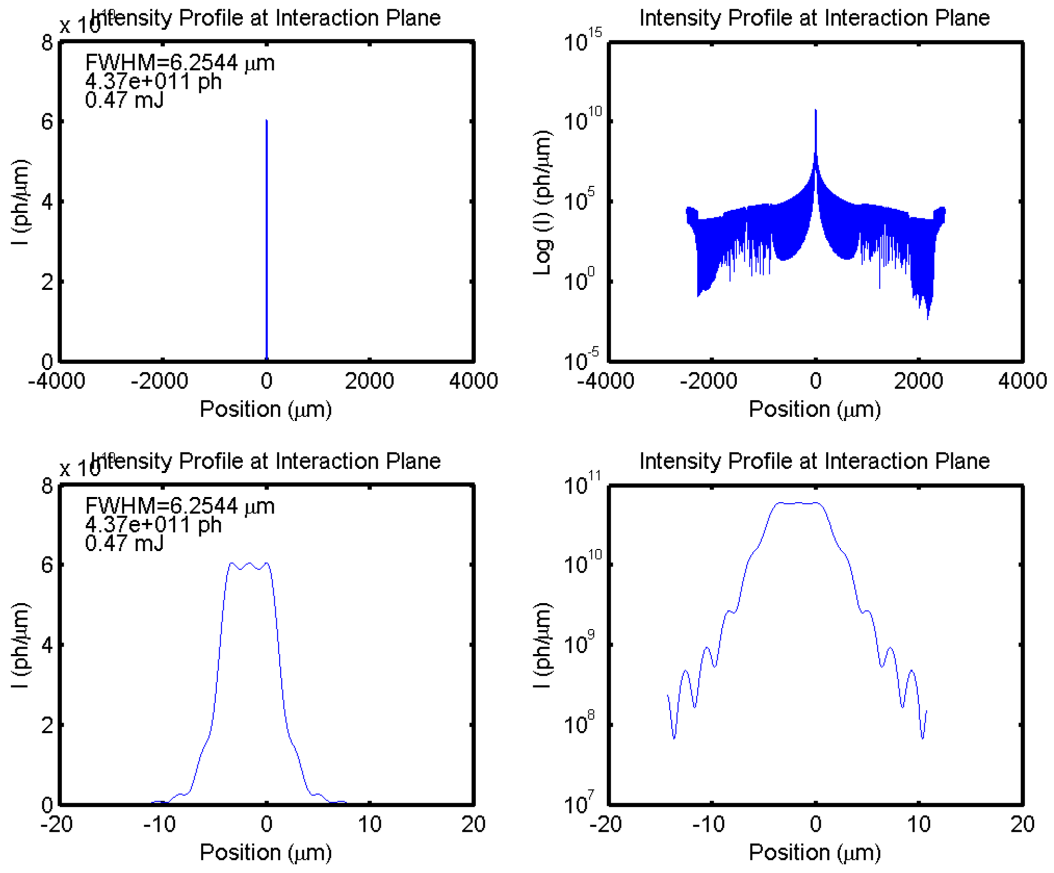

Figure S36 Beam profile at the refocused interaction plane in the horizontal direction for 6.733 keV.

S2.3. HFM with simple simulated HOMS figure error

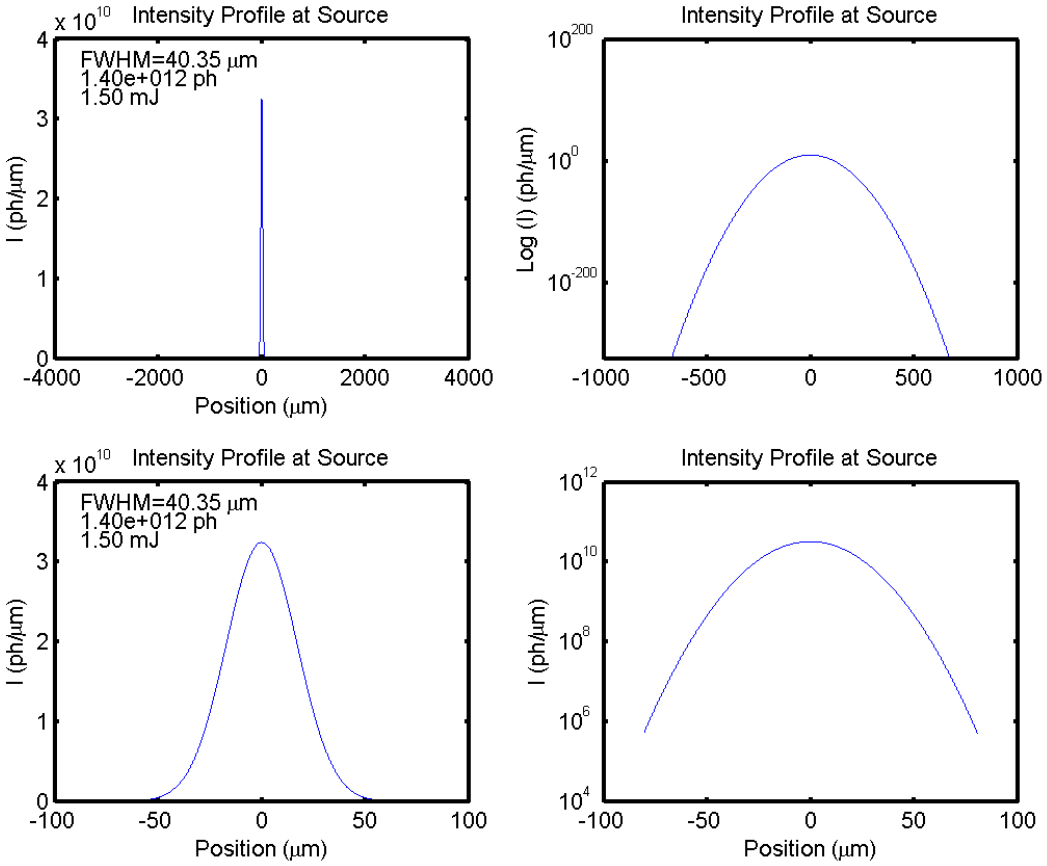

Figure S37 Beam profile at the source in the horizontal direction for 6.733 keV.

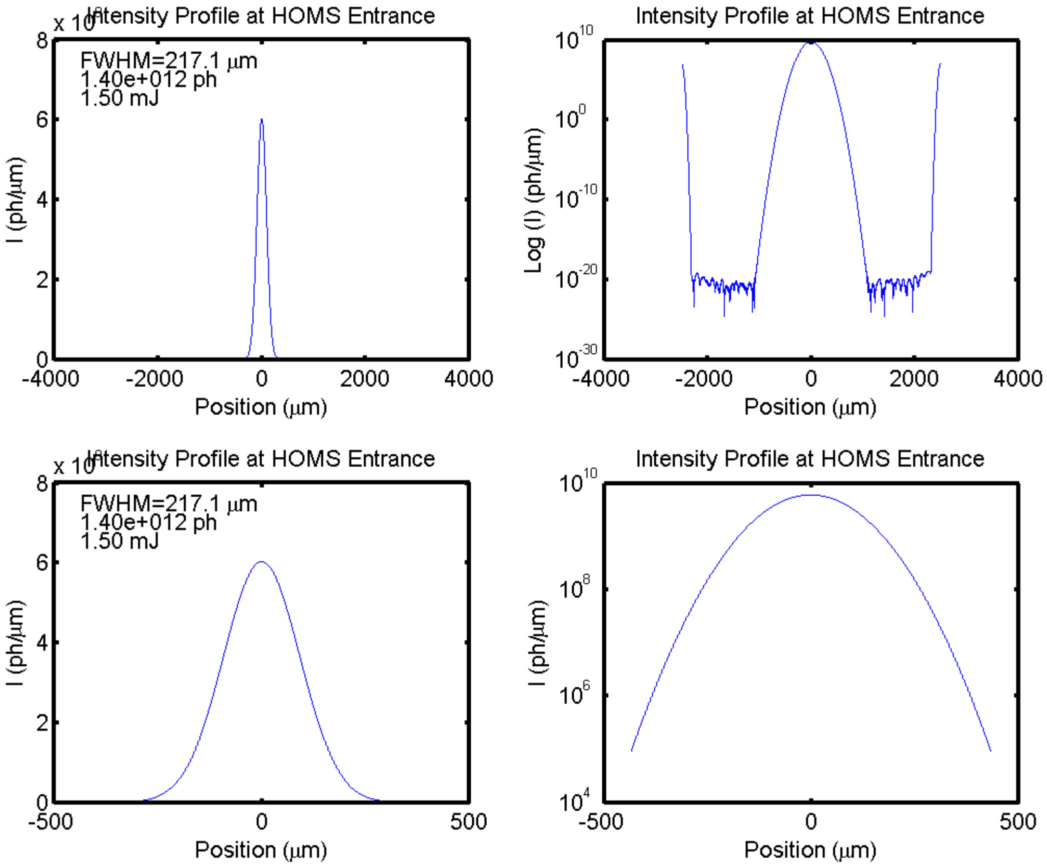

Figure S38 Beam profile at the HOMS entrance in the horizontal direction for 6.733 keV.

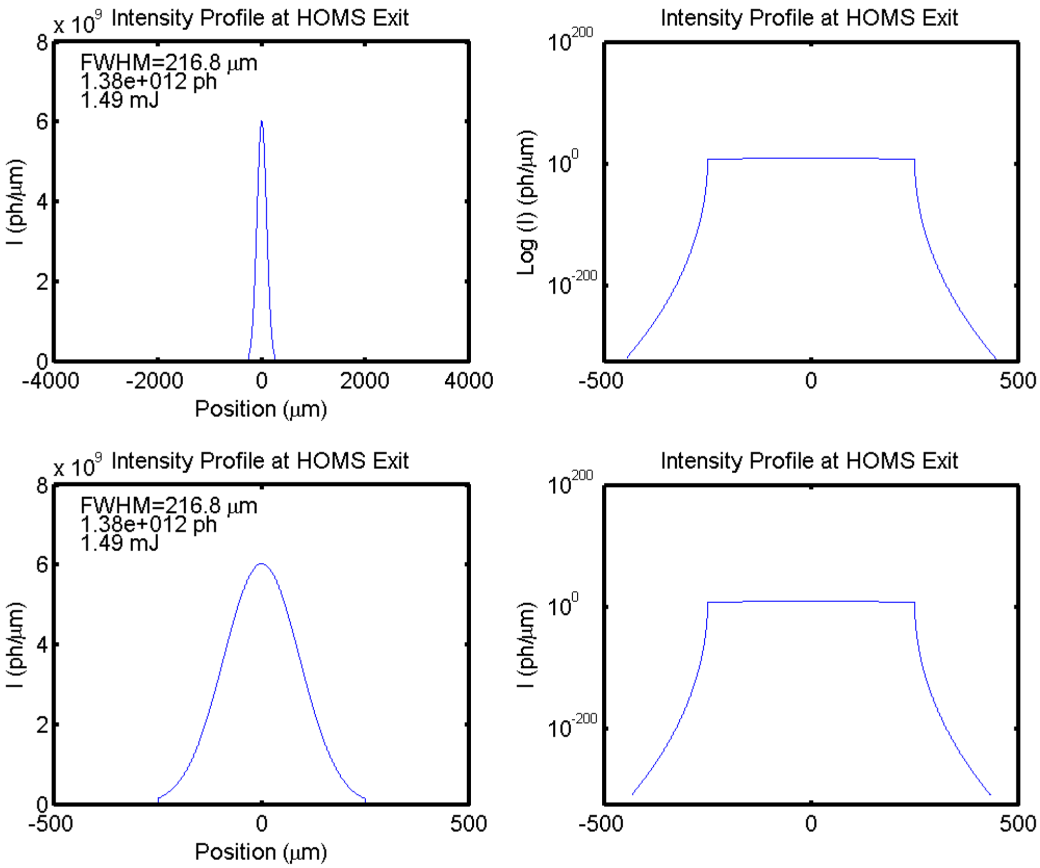

Figure S39 Beam profile at the HOMS exit in the horizontal direction for 6.733 keV.

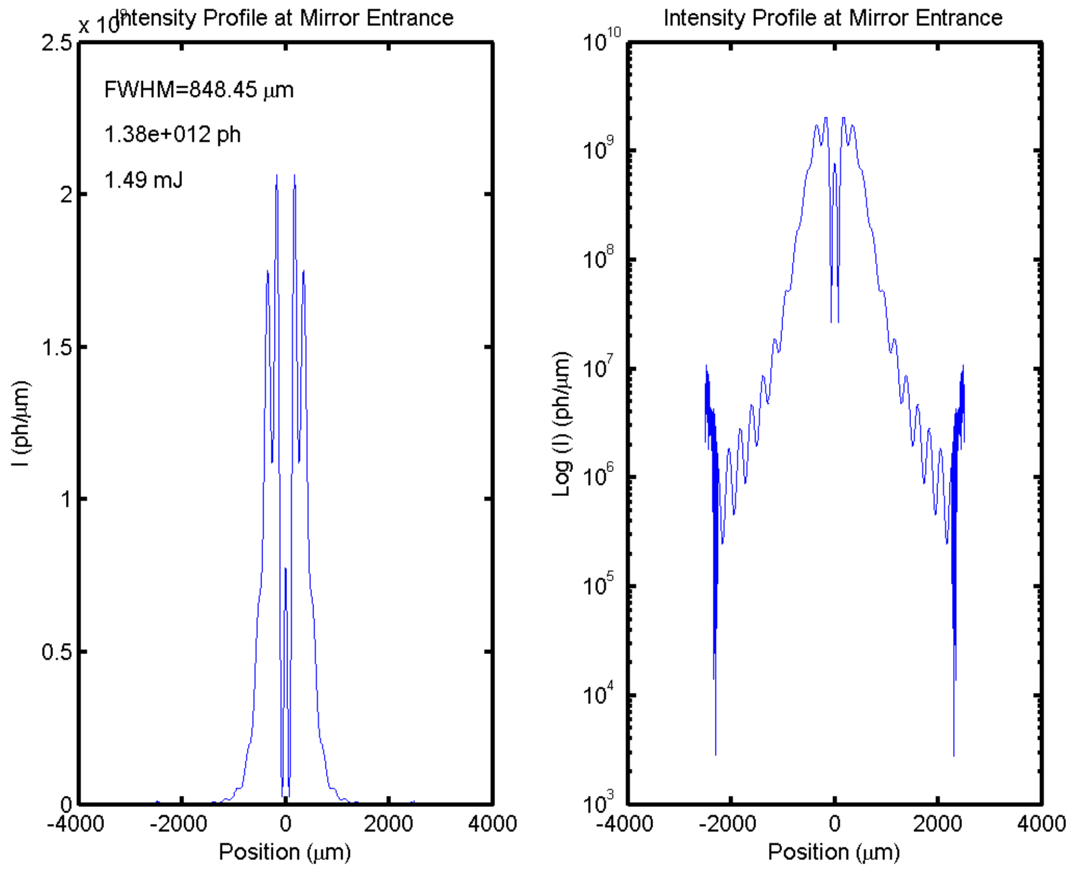

Figure S40 Beam profile at the HFM entrance in the horizontal direction for 6.733 keV.

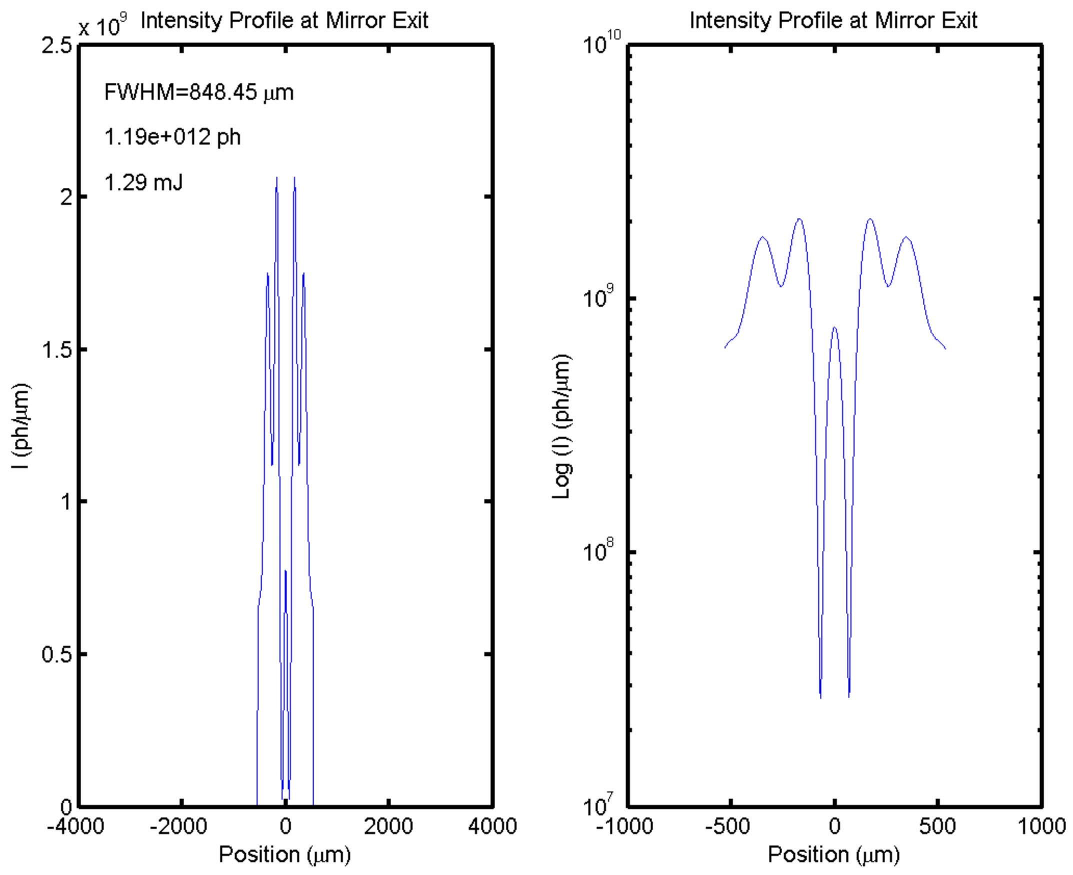

Figure S41 Beam profile at the HFM exit in the horizontal direction for 6.733 keV.

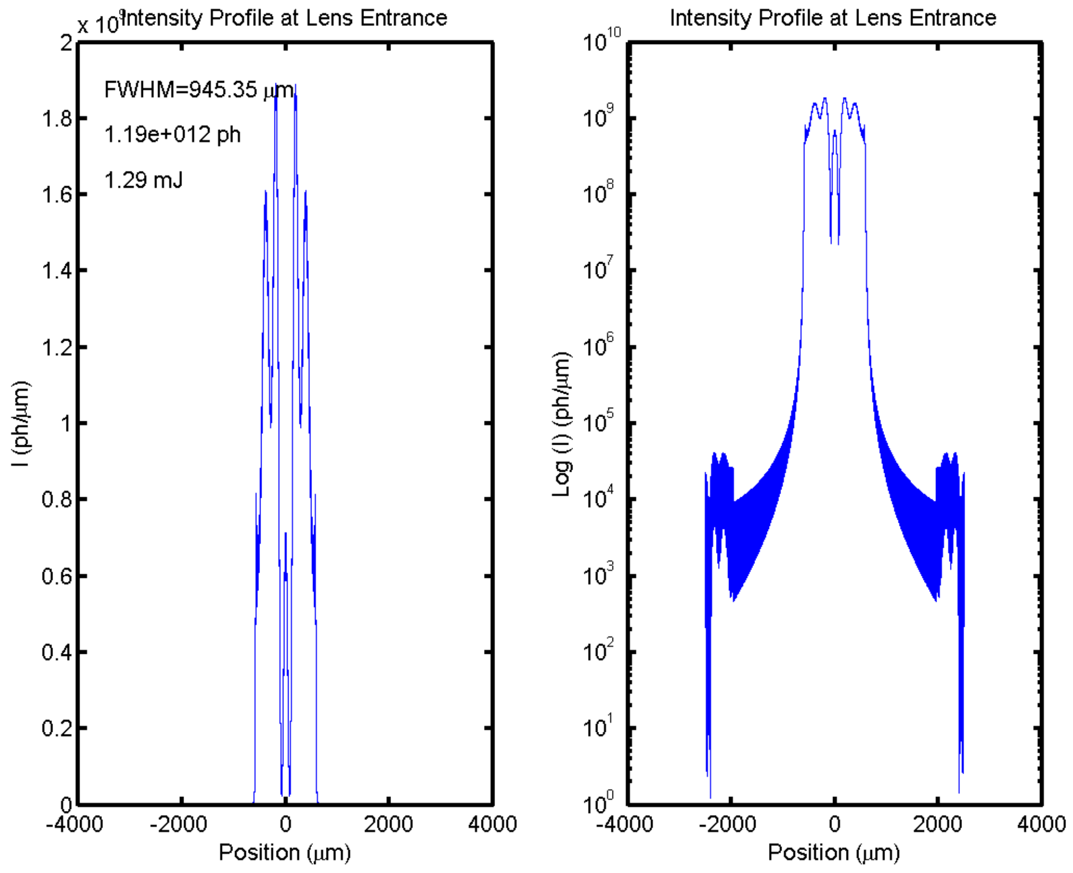

Figure S42 Beam profile at the Be lens entrance in the horizontal direction for 6.733 keV.

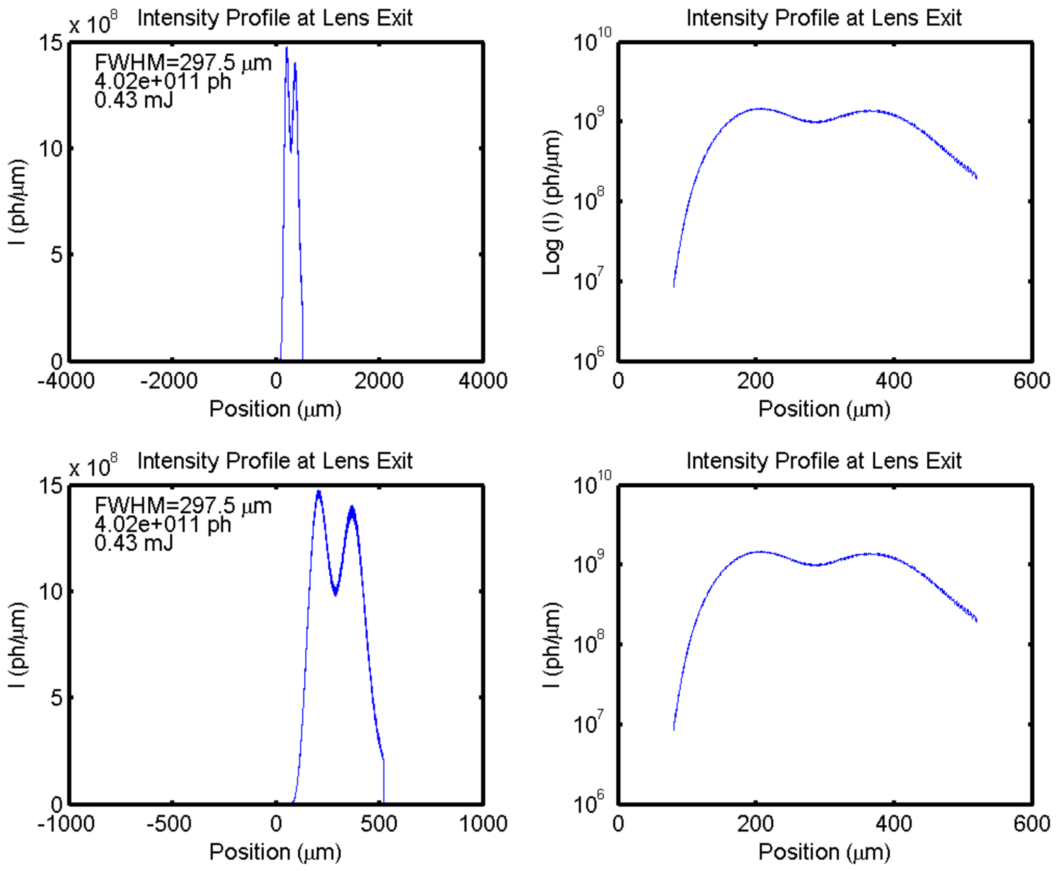

Figure S43 Beam profile at the Be lens exit in the horizontal direction for 6.733 keV.

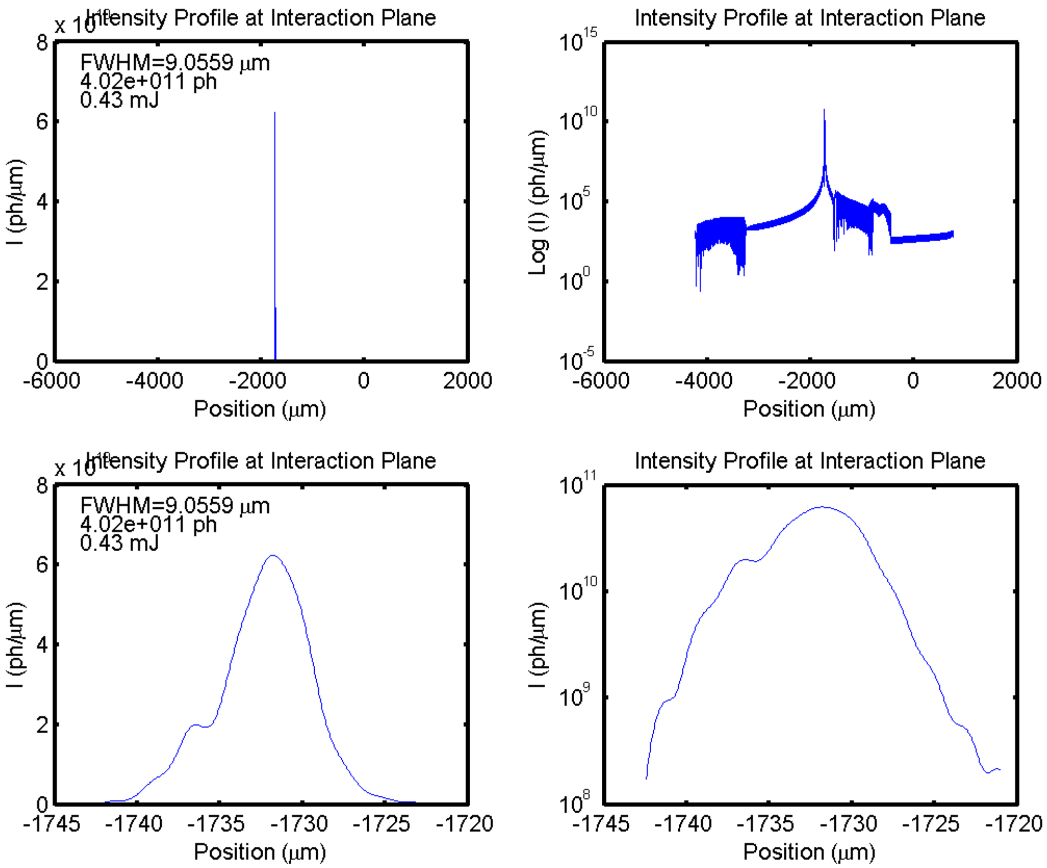

Figure S44 Beam profile at the refocused interaction plane in the horizontal direction for 6.733 keV.
